# Antioxidant activity and selective cytotoxicity in HCT-116 and WI-38 cell lines of LC-MS/MS profiled extract from *Capparis spinosa L*

**DOI:** 10.3389/fchem.2025.1540174

**Published:** 2025-04-10

**Authors:** Amjad Ibrahim Oraibi, Ashour H. Dawood, Ghada Trabelsi, Ousman B. Mahamat, Leila Chekir-Ghedira, Soumaya Kilani-Jaziri

**Affiliations:** ^1^ Department of Pharmacy, Al-Manara College for Medical Sciences, Maysan, Iraq; ^2^ Department of Pharmaceutical Sciences A, Faculty of Pharmacy of Monastir, University of Monastir, Monastir, Tunisia; ^3^ Research Laboratory for Bioactive Natural Products and Biotechnology LR24ES14, Faculty of Dental Medicine of Monastir, University of Monastir, Monastir, Tunisia; ^4^ Department of Pharmaceutical Sciences, Al-Esraa University, Baghdad, Iraq; ^5^ National Federation Associations of Healers and Practitioners of Medicine, the Ministry of Public Health of Chad, N’Djamena, Chad

**Keywords:** *Capparis spinosa* L., antioxidant activity, rutin, resveratrol, MTT assay

## Abstract

**Introduction:**

*Capparis spinosa* L. is significant among the family Capparidaceae for its survival and tolerance to dry environments. In this study, we evaluate the antioxidant and anticancer activities of extracts of roots and aerial parts of *Capparis spinosa* L.

**Methods:**

Bioactive compounds, including phenolic acids and flavonoids, in various ethyl acetate fractions from the extracted roots and aerial parts, were identified using LC-MS/MS. Principal leaf constituents characterized included Rutin, Resveratrol, Astragalin, and others. The Rutin, Resveratrol, Astragalin, (of ethyl acetate fraction), leaves, and roots were screened for antioxidant activity using DPPH, FRAP, ABTS, and CUPRAC activity assays, as well as for cytotoxicity with the MTT assay.

**Result:**

The antioxidant and anticancer activities of the samples were evaluated using DPPH, ABTS, FRAP, CUPRAC assays, and the MTT assay. Roots and Rutin consistently exhibited the strongest antioxidant activity across all assays, with Roots (IC_50_ = 0.06–0.36 mg/mL) excelling in FRAP and CUPRAC, and Rutin (IC_50_ = 0.013 mg/mL) showing the highest DPPH activity. In contrast, Astragalin displayed the weakest antioxidant potential. For anticancer activity, the MTT assay revealed that Leaves (IC_50_ = 23.26 μg/mL) and Roots (IC_50_ = 34.65 μg/mL) were the most potent against HCT-116 cells, outperforming Nutlin (IC_50_ = 62.72 μg/mL), with minimal toxicity to normal WI-38 cells. These results highlight the therapeutic potential of Roots and Rutin as strong antioxidant and anticancer agents.

**Conclusion:**

The results provide useful information concerning the medicinal potentials of *Capparis spinosa* L., particularly about HCT-116 and WI-38 cell line selectivity, and its relevance in the synthesis of natural antioxidants.

## 1 Introduction

The *Capparis spinosa* L. is significant among the family Capparidaceae for its survival and tolerance to dry environments. Although not intensively cultivated, *C. spinosa* is grown in many places worldwide including the Mediterranean basin (Levizou et al., 2004; Sun et al., 2023). Furthermore, *C. spinosa* not only contributes to the Mediterranean diet but has also been used to treat various ailments in the traditional herbal medicine practice because of its tonic, diuretic, and antihypertensive actions (Sun et al., 2023). Different forms of *C. spinosa* are said to be beneficial when used to mitigate the challenges faced by mankind like hypolipidaemic and anti-diabetic effects (Mollica et al., 2017; Shahrajabian et al., 2021). Studies on the methanol extracts of *C. spinosa* herb had antioxidant properties and free radical scavenging properties *in vitro* which would be beneficial in conditions related to oxidative stress (Bacchetti et al., 2022; Grimalt et al., 2022). Earlier works have focused on the beneficial components of *C. spinosa* such as vitamins, flavonoids, and polyphenols which dispense antioxidant properties to the products (Mollica et al., 2017; Shahrajabian et al., 2021; Annaz et al., 2022).

Usually, both leaves and flower buds are high in Flavonoids and Phenolic substances that are the most potential antioxidants (Grimalt et al., 2022). Besides the antioxidant effect, *C. spinosa* has been reported in abundance in traditional medicine for antispasmodic, analgesic, diuretic, and expectorant properties. It has also been used for treating many conditions like curing inflammation and gastrointestinal disorders, treating anemia and liver diseases, and relieving rheumatic pain (Gull et al., 2015; Rahimi et al., 2020; Sun et al., 2023). Even within a particular region, different plant parts, such as the young shoots and other plant samples, have been incorporated into traditional medicine practices. In Greece, infusion of the young shoots was taken for relief in rheumatism, and in Libya, they focused on the tumor-curing properties of these young shoots (Rivera et al., 2003). In Syria, vinegar preparation of dried leaves was used to apply on head scabs and ulcers (Elachouri et al., 2024). The apical flower buds were utilized for respiratory problems, kidney stones, and gastrointestinal problems (Abdulridha and Saliem, 2023; Yang et al., 2023). Several researchers in different countries have studied the sections of *C. spinosa* for their antioxidant activity (Allaith, 2016). These activities are normally attributed to the high amounts of flavonoids and phenolic acids present in this plant (Alqahtani et al., 2023). These metabolites are important in understanding the medicinal aspects of the plant as well as the bioactive compounds that are responsible for the therapeutic activities. The screening employed many qualitative tests to screen for flavonoids, alkaloids, coumarins, terpenes, phenolic compounds, saponins, tannins, and cardiac glycosides. Such secondary metabolites are important they could possess valuable antioxidant, anti-inflammation, and antibacterial activities (Nazer et al., 2021).

The objective of this article is to investigate some bioactive compounds obtained from *Capparis Spinosa* L. (root and leaves), rutin, resveratrol, and astragalin for Free radical scavenging properties and anticancer effect.

## 2 Material and method

### 2.1 Plant material

The plant parts of Capparis Spinosa L. were collected from the Maysan region in southern Iraq, where it naturally grows. All plant parts were thoroughly washed and kept for drying in the shade for 3 weeks. Once dried, the plant material was ground into a fine powder using an electric grinder.

### 2.2 Experimental work

The experimental procedures were divided into the following steps.

#### 2.2.1 Extraction and fractionation of leaves and stem (Soxhlet extraction)

For the extraction process, 400 g of the powdered plant material (leaves and stem) were soaked in 1,500 mL of petroleum ether (boiling point 30°C–60°C) to remove non-polar materials. The mixture was shaken frequently over 3 days. After filtering, the petroleum ether extract was set aside for further analysis, and the remaining plant material was spread on paper to evaporate any remaining solvent. The dried plant powder was then subjected to Soxhlet extraction using 80% hydroethanolic Solvent (a solution prepared with 80% ethanol and 20% water) for 12 h. The resulting extract was filtered, and the filtrates were combined. The solvent was removed under vacuum using a rotary evaporator, yielding a dark greenish residue. This residue was suspended in 500 mL of water and successively partitioned with chloroform, ethyl acetate, and n-butanol (3 × 500 mL for each fraction) using a separating funnel. The chloroform and ethyl acetate fractions were dried over anhydrous sodium sulfate, filtered, and evaporated to dryness using a rotary evaporator. The scheme of extraction is shown in Supplementary Figure S1.

#### 2.2.2 Extraction and fractionation of roots (Soxhlet extraction)

In order to extract the roots, 150 g of powdered Capparis spinosa root were soaked in 500 mL of petroleum ether for 3 days to remove non-polar compounds. During this process, the mixture was shaken at intervals. After 3 days, the petroleum ether was separated by filtration, and the filtrate was removed from the filter paper and taken away for some hours to allow evaporation. After the hydroethanolic extract was concentrated, the solid plant residue was further liquidated by 80% ethanol for 12 h in a soxhlet thimble. The hydroethanolic extract was filtered, and the solvent was evaporated under vacuum using a rotary evaporator. This residue was dissolved in water 250 mL and further separated into several portions using chloroform, ethyl acetate, and n-butyl alcohol fractions as in the previous work for leaves and stem fractions. The fractions of petroleum ether, chloroform, ethyl acetate, and n-butanol were collected and preserved for further evaluation. Schematic diagram for fractionation of roots of *Caparis spinosa* crude extracts is shown in Supplementary Figure S2.

### 2.3 Preliminary phytochemical screening of aerial parts and roots

Phytochemical Investigation focused mostly on the presence and/or absence of various secondary metabolites in the aerial parts and the roots of *Capparis spinosa* L. and has used quite several tests. These tests aimed to slope out selected materials, particularly plant flavonoids, phenolic acids, plant alkaloids, and other plant materials.

### 2.4 LC-MS/MS analysis of ethyl acetate fractions

Advanced liquid chromatography-mass spectrometry (LC-MS) techniques were employed to identify and confirm the presence of flavonoids and phenolic acids within the ethyl acetate fractions derived from the aerial parts and roots. This method was employed to isolate and characterize the polar compounds from the ethyl acetate fractions and also improved the understanding of the chemical composition of these extracts.

### 2.5 Chromatographic analysis LC-MS/MS and GC-MS

#### 2.5.1 LC-MS/MS analysis

##### 2.5.1.1 Instrumentation

The analysis was performed using a liquid chromatography system (ATLAS_QTOF_ICX_V0 O4, Germany) with an Integrated ExionLC 3.61 pump system (ACCBM5671761) and a Valve model 1.0.0.0 (AB SCIEX 1). A C18–ODS column (250 mm × 4.6 mm i.d., 5 μm particle size) was used for separation.

##### 2.5.1.2 Conditions

Ionization Mode: Negative ion mode.

Mass Range: Full scan spectra (m/z 50–900) with MS/MS fragmentation on selected ions.

Nebulization & Drying: High-purity nitrogen at optimized temperatures and flow rates.

Detector: UV at 280 nm.

##### 2.5.1.3 Mobile phase

The mobile phase was composed of:

Solution A: Methanol.

Solution B: 0.05% Trifluoroacetic acid (TFA) in water.

##### 2.5.1.4 Gradient elution

The separation was carried out using a gradient elution at a constant flow rate of 1.0 mL/min, as follows:

**Table:** 

Step	Time (min)	A (%) (Methanol)	B (%) (0.05% TFA)
0	0	40	60
1	5	70	30
2	15	90	10
3	20	90	10
4	25	40	60
5	30	40	60

##### 2.5.1.5 Data analysis

The acquired LC-MS/MS data were processed using Analyst 1.6.3 software (Germany-Darmstadt) for compound identification and structural elucidation (El-Elimat et al., 2024).

#### 2.5.2 GC-MS analysis of chloroform and petroleum ether extracts

The GC-MS analysis of petroleum ether and chloroform fractions from both the aerial parts and roots of Capparis spinosa was conducted using an Agilent GC-MS system, equipped with an Agilent 190915-433UI HP-5ms Ultra Inert column.

Instrument Setup and Operating Conditions:

Column: Agilent 190915-433UI (HP-5ms Ultra Inert).

Injection Mode: Front Split/Splitless (SSZ).

Carrier Gas: High-purity helium (99.995%).

Flow Rate: 0.9 mL/min.

Carrier Gas Pressure: 7.037 psi.

Average Linear Velocity: 34,772 cm/s.

Hold-Up Time: 1.4379 min.

Temperature Program:

Initial Oven Temperature: 60°C.

Temperature Ramp 1: Increase from 60°C to 150°C at 3°C/min, hold for 10 min.

Temperature Ramp 2: Increase from 150°C to 300°C at 10°C/min.

Detection and Data Analysis:

Detector: Mass Selective Detector (MSD).

Ionization Mode: Electron Ionization (EI) at 70 eV.

Sample Injection: 1 µL of 1% extract solution (diluted in respective solvents) was injected in splitless mode.

Compound Identification: Based on mass spectral fragmentation patterns using NIST and Wiley spectral libraries.

Quantification: Relative abundance of detected compounds was expressed as a percentage based on peak area in the chromatogram (Seetharaman et al., 2025).

### 2.6 Antioxidant and free radical scavenging activity

#### 2.6.1 1, 1-diphenyl-2-picrylhydrazyl radical antioxidant study

Antioxidant properties of the aerial parts, roots, resveratrol, astragalin, and rutin fractions were assessed using the DPPH radical scavenging assay. DPPH assay was performed using the following procedure (Brand-Williams et al., 1995; Baliyan et al., 2022): An amount of 0.5 mg DPPH was dissolved in 10 mL of ethanol to give a 1 mg/mL DPPH stock concentration. This solution was stored in a dark glass bottle to avoid proper temperature conditions and/or sunlight to keep it stable throughout the experiment. Four sample solutions were prepared with the following concentrations: 1 mg/mL, 0.1 mg/mL, 0.01 mg/mL, and 0.001 mg/mL in ethanol. These concentrations of the samples made it possible to use the scavenging activity of each of the samples over a range of dilutions. The DPPH assay was performed in a 96-well microplate, which included the addition of 200 µL of DPPH solution in each well as a source of free radicals. Later, 100 µL of different sample solutions at different concentrations was added in the respective wells after the DPPH solution was added. As a control, 100 µL of ethanol was placed instead of the sample in separate wells to assess the DPPH absorbance without the addition of any antioxidant. Further blanks were carried out using 200 µL of ethanol instead of DPPH solution along with 100 µL of each of the samples, to take care of the probable absorbance of the samples themselves ethanol. Mixtures were then incubated in the dark at room temperature for 30 min to allow the reaction of the DPPH radicals with possible antioxidants in the samples in solution. Following this period, the absorbance of each of the mixtures was measured at 517 nm using a UV-Vis microplate reader. The percentage of DPPH radical scavenging activity was calculated using the following formula:

$$\text{DPPH Scavenging Activity }\left(\%\right)=\left(A\text{ control }–A\text{ sample }/\;A\text{ control}\right)\times 100$$

In this formula, A control refers to the absorbance of DPPH solution with ethanol whereas A sample refers to the absorbance of DPPH solution absorbed in the sample. In this manner, a calculation of the percentage of DPPH radical scavenging activity for each sample was done along with IC_50_. The lower the IC_50_ value of the samples, the greater the antioxidant activity since it conveyed that less of the sample was needed to reduce the DPPH radicals by 50%. The DPPH scavenging effects of the aerial part, roots extract, resveratrol, astragalin, and rutin were analyzed based on their IC_50_ or extent of activity at different concentrations. They were also compared to assess their DPPH free radical scavenging ability in depth and anti-oxidative capacity as well (Moukette et al., 2015).

#### 2.6.2 ABTS assay

The antioxidant activities of the aerial part of the plant, root, resveratrol, astragalin, and rutin were evaluated using the ABTS radical cation (ABTS⋅⁺) assay. The ABTS⋅⁺ solution preparation method was done according to (Ilyasov et al., 2020) which includes:39.2 mg ABTS dissolved in distilled water and subsequently completed with a 6.7 mM potassium persulfate solution in equal volume (We Prepared a 6.7 mM potassium persulfate solution by dissolving 17.6 mg of potassium persulfate in 5 mL of distilled water.). The solution was allowed to stand in the darkroom at a temperature for 12–16 h to form the ABTS radical cation. This resulting solution was then further diluted with ethanol to measure the maximum probable absorbance density of about 0.70 (±0.02) at 734 nm. Trolox standards were prepared in ethanol from 0.01 to 0.1 mM and sample solutions were prepared in ethanol at required concentrations. In the assay, 200 µL sample solution was taken in each cuvette, and 2 mL ABTS⋅⁺ solution was added to each tube or cuvette for mixing. Controls were prepared by mixing 2 mL of ABTS⋅⁺ solution with 200 µL of ethanol, while blanks were prepared by mixing 2 mL of ethanol with 200 µL of each sample solution (without ABTS⋅⁺). The mixtures were incubated in the dark at room temperature for 30 min before the absorbance was measured at 734 nm using a UV-Vis spectrophotometer. The percentage of ABTS radical scavenging activity was calculated using the following formula:

$$\text{ABTS Scavenging Activity }\left(\%\right)=\left(A\text{ control}-A\text{ sample }A\text{ control }\right)\times 100$$

Where A control is the absorbance of the ABTS⋅⁺ solution with ethanol, and A sample is the absorbance of the ABTS⋅⁺ solution with the sample. IC_50_ was determined by plotting the percentage of scavenging activity against the concentrations of the samples; IC_50_ is the concentration at which it shows 50% scavenging of the ABTS radicals. Based on IC_50_ values, and for some samples at definite concentrations, the antioxidant activities of the five tested samples were compared (Rumpf et al., 2023).

#### 2.6.3 Ferric reducing antioxidant power assay

The ferric reducing antioxidant power (FRAP) assay was selected to evaluate the antioxidant activity of the aerial part, root, and fractions of resveratrol, astragalin, and rutin. For this assay, the reagents were prepared according to Skroza et al. (2015) with some modifications: The acetate buffer was prepared by adding approximately 16 mL of glacial acetic acid to 3.1 g of sodium acetate trihydrate and volumetrically making up to 1 L with distilled water. A TPTZ solution was prepared by dissolving 0.031 g in 10 mL of 40 mM HCl and a ferric chloride solution was prepared by dissolving 0.054 g of FeCl_3_·6H_2_O in 10 mL of distilled water. The FRAP working solution was made up immediately before use by mixing acetate buffer, TPTZ solution, and ferric chloride solution in a ratio of 10:1:1. Sample solutions from root extract, leaf extract, resveratrol, astragalin, and rutin were prepared in ethanol at a concentration of choice. The assay was carried out by first dispensing 180 µL of the FRAP working solution into each well of a 96-well microplate before adding 20 µL of each sample solution or trolox standard to the wells. The plate was then incubated for half an hour at 37°C before the absorbance reading was undertaken at 593 nm using a microplate reader. This absorbance was employed to estimate the reducing power of samples according to their capability to convert Fe³⁺ to Fe^2^⁺ in the presence of TPTZ (Benzie and Devaki, 2018).

#### 2.6.4 Cupric reducing antioxidant capacity assay

Cupric reducing antioxidant capacity (CUPRAC) assay was utilized for screening additional radical scavenging activity of the aerial part, root, resveratrol, astragalin, and rutin. To perform the CUPRAC assay the following reagents were prepared according to Apak et al. (2010) 0.4262 g of copper (II) chloride dihydrate (CuCl_2_·2H_2_O) was dissolved in 200 mL of distilled water to make a 0.01 M copper (II) chloride solution. For preparing 1 M ammonium acetate buffer (pH 7.0), 7.708 g of ammonium acetate was dissolved in 100 mL of distilled water, and about 60 mL of this buffer was supplemented with 0.0075 M neocuproine solution prepared by dissolving 0.039 g of neocuproine in 20 mL of ethanol. Trolox standard solutions were prepared in ethanol with concentrations ranging from 0.01 to 0.1 mM. Sample solutions from root extract, leaf extract, resveratrol, astragalin, and rutin were also prepared in ethanol at desired concentrations. For the assay, 150 µL of the pre-mixed reagent solution [copper (II) chloride, ammonium acetate, and neocuproine] was pipetted into each well of a 96-well microplate, followed by the addition of 50 µL of each sample solution or standard Trolox solution. The microplate was incubated at room temperature for 30 min in the dark to allow the reaction to occur. The absorbance was measured at 450 nm using a UV-Vis microplate reader. The antioxidant activity was calculated using the formula:

$$\text{CUPRAC Activity }\left(\%\right)=\left(\;A\text{ sample }–\;A\text{ blank }/\;A\text{ control }\right)\times 100$$

Where A sample is the absorbance of the sample solution, A blank is the absorbance of the blank (without CUPRAC), and A control is the absorbance of the Trolox standard. The IC_50_ value was determined by plotting the CUPRAC activity percentage against the sample concentrations where this value represents the concentration necessary to achieve 50% of the maximum CUPRAC activity observed from the standard. The antioxidant activities of the five samples were also compared based on IC_50_ values or CUPRAC activities at a given concentration (Suktham et al., 2019).

### 2.7 *In vitro* MTT (3-[4, 5-dimethylthiazol-2-yl]-2, 5) diphenyl tetrazolium bromide) cell proliferation assay

#### 2.7.1 Cell culture and media

Cell lines were purchased from the Holding Company for Biological Products and Vaccines (VACSERA, Giza, Egypt). HCT-116 and WI-38 cells were cultured in RPMI-1640 medium. Enhanced with 10% fetal bovine serum (FBS) and 1% penicillin-streptomycin mixture (100 IU/mL penicillin and 0.1 mg/mL streptomycin) (Calmeiro et al., 2021).

#### 2.7.2 Procedure

MTT (3-(4,5-dimethylthiazol-2-yl)-2,5-diphenyl tetrazolium bromide; Sigma) was dissolved in PBS at a concentration of 5 mg/mL and filtered to sterilize and remove a small amount of insoluble residue present in some batches of MTT. At the times indicated below, the original MTT solution (10/∼1 per 100/∼1 medium) was added to all test wells and the plates were incubated at 37°C for 4 h. Isopropanol acid (100/∼1 of 0.04 N HCI in isopropanol) was added to all wells and mixed well to dissolve the dark blue crystals. After a few minutes at room temperature to ensure all crystals were dissolved, the plates were read on a Dynatech MR580 Microelisa Reader, using a test wavelength of 570 nm, a reference wavelength of 630 nm, and a calibration setting of 1.99 (or 1.00 if the samples were strongly colored). Plates were typically read within 1 h of adding isopropanol.

#### 2.7.3 *In vitro* MTT cell proliferation assay

The MTT assay was used to evaluate the proliferation of control and treated cells according to Ghasemi et al. (2023). 2.6∼3 × 10^4^ cells were added to each well of a 96-well tissue culture plate containing the appropriate media and grown for 24 h. Drug stock solutions were prepared in DMSO. Eight concentrations (300, 100, 30, 10, 3, 1, 0.3, and 0.1 μg/mL) were prepared for each compound in the growth media. The Nutlin was used as a reference compound. Cells were then treated for 72 h. Freshly prepared MTT salt (3-(4,5-dimethylthiazol-2yl)-2,5- diphenyltetrazolium bromide) (5 mg/mL; Sigma) was then added to each well to give a final concentration of 0.5 μg/μL. The plates were incubated for 4 h and the formation of formazan crystals was checked using an inverted microscope. An Equal volume of 1:1 (200 μL) DMSO and isopropanol mixture was added to each well and incubated for 30–45 min. Cell proliferation was detected by measuring the absorbance of each well at 590 nm using Multiskan^®^ EX (Thermo Scientific, United States) Microplate Reader. The Experiment was performed three times in triplicates (Ghasemi et al., 2023).

### 2.8 Statistical study

All methods were conducted in triplicate, and results are expressed as the mean ± standard deviation (SD). Statistical analyses, including one-way ANOVA and multiple group comparisons, were performed using GraphPad Prism version 10.4.1. Statistical significance was set at p < 0.05.

One-way ANOVA analysis of cytotoxic effects on HCT-116 and WI-38 cells

To assess the statistical significance of the cytotoxic effects of the tested compounds on HCT-116 (colon cancer) and WI-38 (normal) cell lines, a one-way analysis of variance (ANOVA) was performed. The ANOVA test was applied to determine whether there were statistically significant differences in cell viability among the different tested samples. Post-hoc analysis using Tukey’s test was conducted when significant differences were found to identify specific group variations.

## 3 Result

### 3.1 Phytochemical screening of crude extracts

The phytochemical analysis of the crude extracts from the aerial parts and roots of *Capparis spinosa* L. was done to establish its major classes of secondary metabolites.

### 3.2 LC-MS/MS and GC-MS analysis: phytochemical investigation of ethyl acetate fractions from aerial parts and roots

**FIGURE 1:**
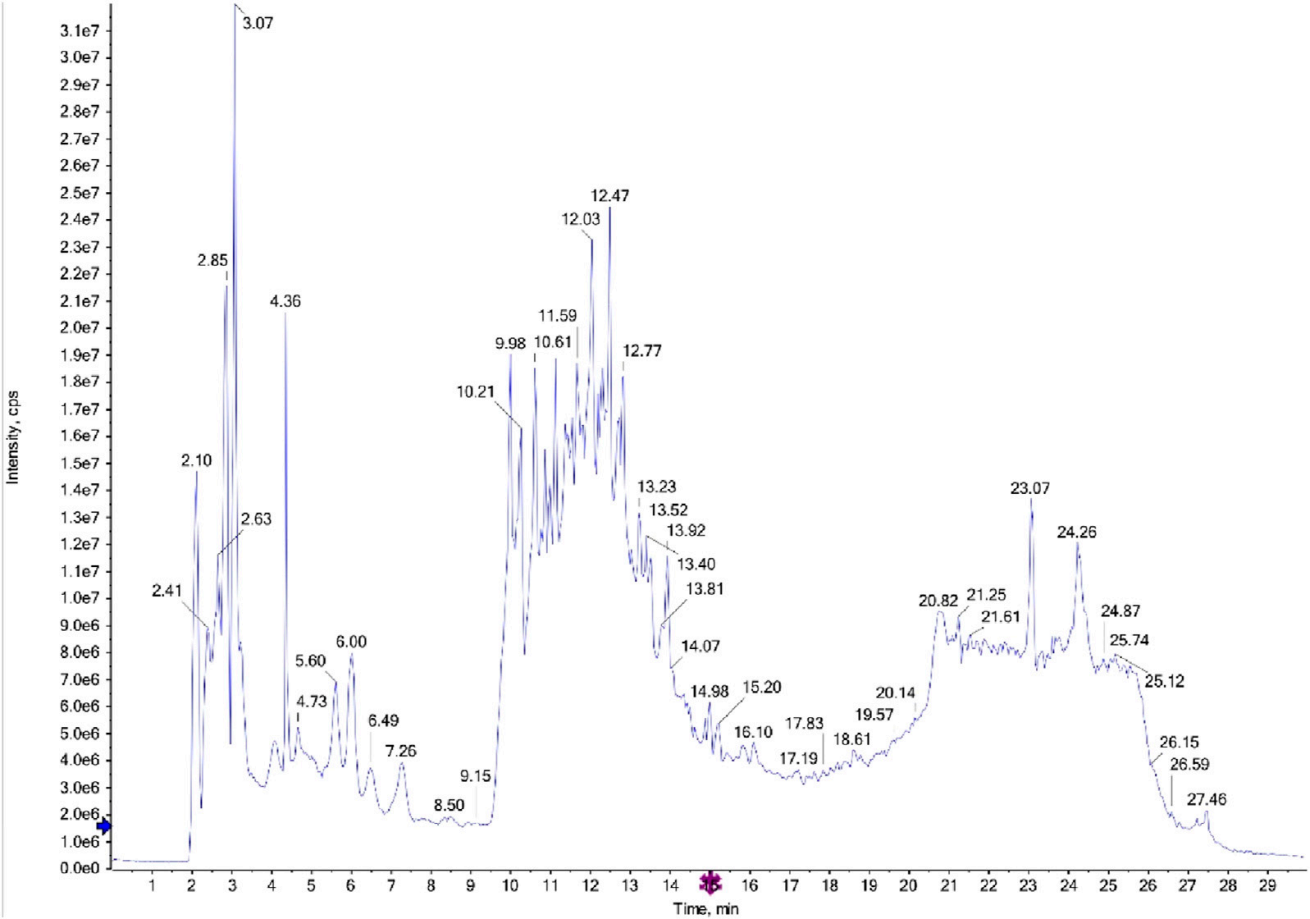
LC-MS/MS diagram of ethyl acetate fraction of the aerial parts.

**FIGURE 2:**
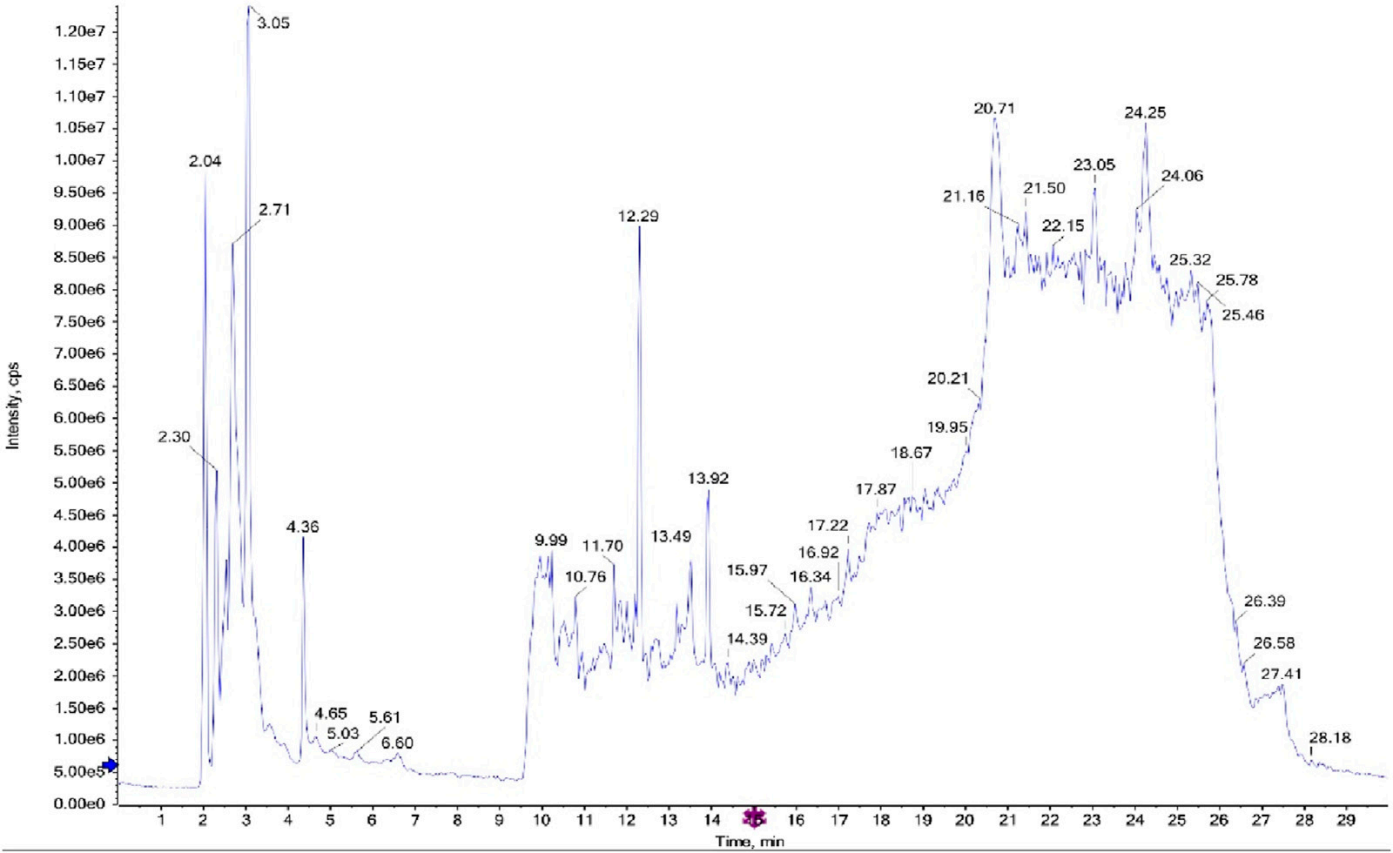
LC-MS/MS diagram of ethyl acetate fraction of the roots.

The LC-MS/MS analysis of the ethyl acetate fractions from the aerial parts and roots revealed the presence of several bioactive compounds, with notable differences between the two fractions. The detailed results, including compound structures and their respective LC-MS/MS spectra, are provided in the Figures 1, 2; Supplementary Tables S1, S2.

A preliminary phytochemical screening of the tested fractions was conducted using GC-MS to identify various bioactive compounds, including flavonoids, phenols, alkaloids, coumarins, cardiac glycosides, and terpenoids. The table and graph are shown in the Supplementary Figures S3–S6; Supplementary Tables S3–S6.

### 3.3 Antioxidant and free radical scavenging activity

The IC_50_ values for the five tested samples Leaves, Roots, Rutin, Astragalin, and Resveratrol, expressed in mg/mL, provide a quantitative measure of the antioxidant capacity of each sample. These results highlight the relative potency of the tested compounds in neutralizing free radicals, with lower IC_50_ values indicating higher antioxidant activity. The data are summarized in Table 1.

**TABLE 1:** This table includes an antioxidant assay of DPPH, FRAP, Cuprac, and ABTS for 5 samples.

Test	Leaves	Roots	Rutin	Astragalin	Resveratrol
DPPH	0.12	0.017	0.013	1.2	0.032
ABTS	0.12	0.11	0.125	1.1	0.1
FRAP	1.4	0.36	1.06	4.08	0.7
CUPRAC	0.7	0.06	0.09	0.126	0.12

Note: The IC_50_ values were determined based on the median response from three independent experiments, with each experiment performed in triplicate to ensure accuracy and reproducibility

#### 3.3.1 DPPH antioxidant results for aerial part, root, rutin, astragalin, and resveratrol

The DPPH method, an established approach for evaluating the free radical scavenging activity, was carried out on the samples in different concentrations (1.0 mg/mL, 0.1 mg/mL, 0.01 mg/mL, and 0.001 mg/mL) in the present study. Supplementary Table S7 also shows the percent inhibition at these concentrations for each of the samples representing all the likely ranges of their antioxidant potentials.

**FIGURE 3:**
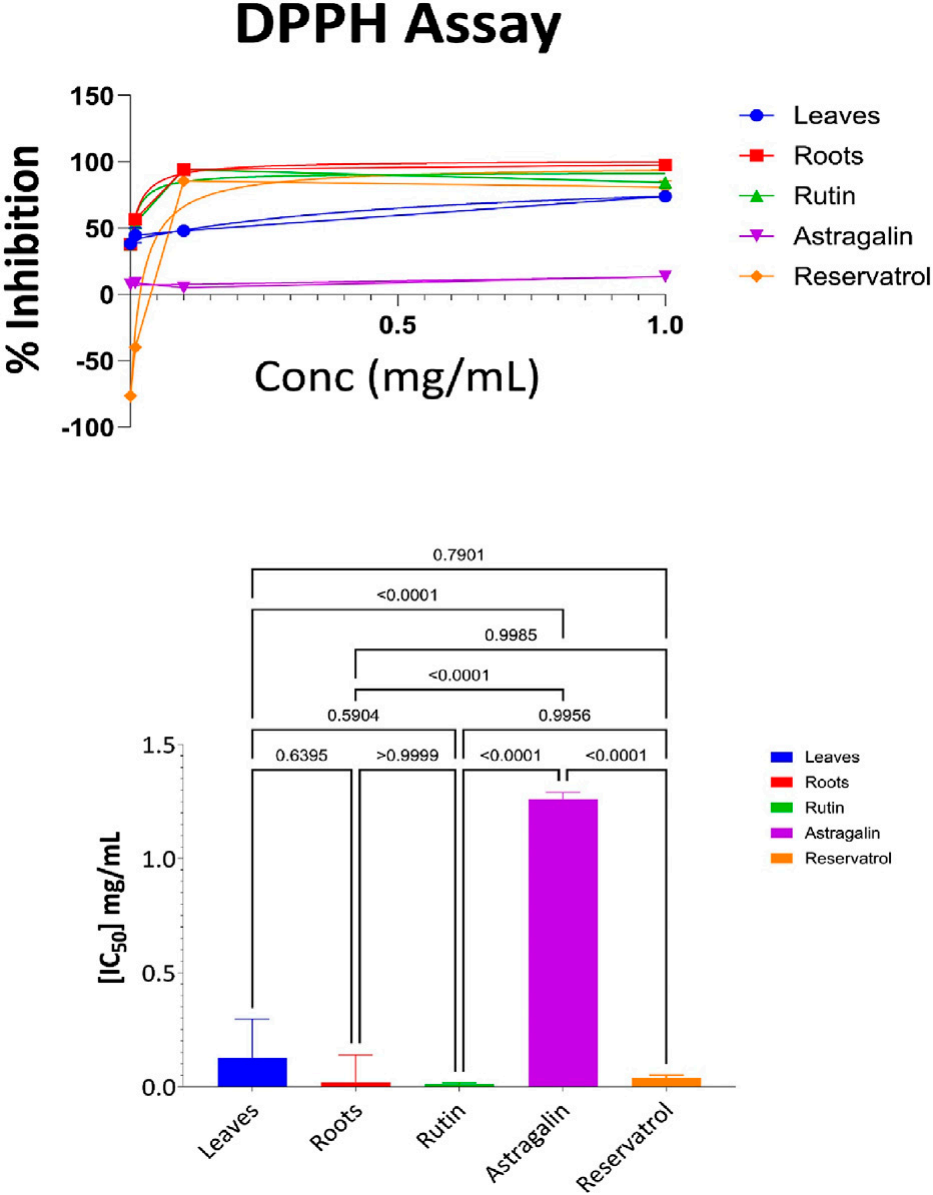
DPPH antioxidant activity: percent inhibition and IC₅₀ with statistical comparisons (One-way ANOVA and Tukey’s test) data are expressed as mean +- SD and n = 3.

As shown in Supplementary Table S7, the root extract exhibited the highest level of antioxidant activity at the highest concentration (97% inhibition at 1 mg/mL). Rutin also showed strong antioxidant activity, achieving 93% inhibition at 0.1 mg/mL and 84% at 1 mg/mL. Resveratrol followed closely, with 77% inhibition at 1 mg/mL and 84% at 0.1 mg/mL. The aerial parts demonstrated moderate activity, with 75% inhibition at the highest concentration. Astragalin, however, showed the weakest activity, with only 11% inhibition at 1 mg/mL and progressively lower values at lower concentrations. The IC_50_ value represents the concentration required to inhibit 50% of the DPPH radicals. Lower IC_50_ values indicate higher antioxidant potency, as less compound is required to achieve significant inhibition (Djeghim et al., 2024). The IC_50_ values for each sample were determined from the data and are depicted in Figure 3. The DPPH radical scavenging assay was conducted to evaluate the antioxidant activity of the tested samples. The IC_50_ values indicate the concentration required to scavenge 50% of DPPH radicals, with lower values representing higher antioxidant potential. Among the tested compounds, Rutin (IC_50_ = 0.013 mg/mL) and Roots (IC_50_ = 0.017 mg/mL) exhibited the strongest antioxidant activity, followed by Resveratrol (IC_50_ = 0.032 mg/mL) and Leaves (IC_50_ = 0.12 mg/mL), while Astragalin displayed the weakest activity (IC_50_ = 1.2 mg/mL). Statistical analysis using one-way ANOVA and Tukey’s multiple comparison tests confirmed that the differences in antioxidant potential were statistically significant (p < 0.0001) between Astragalin and all other compounds, indicating its significantly lower free radical scavenging capacity. However, no significant differences were observed between Roots and Rutin (p > 0.9999), Leaves and Roots (p = 0.639), or Leaves and Resveratrol (p = 0.791), suggesting comparable antioxidant activity among these compounds. These results highlight the strong radical scavenging properties of Rutin and Roots, while Astragalin exhibited significantly lower antioxidant potential compared to the other samples.

#### 3.3.2 ABTS antioxidant results for aerial part, root, resveratrol, astragalin, and rutin

The further evaluation of the antioxidant properties of the aerial parts, roots, and Resveratrol, Astragalin, and Rutin fractions was carried out using the ABTS radical cation method, which is often used in the estimation of the free radical scavenging potential of the plant. Supplementary Table S8 depicts the percentage of ABTS radicals that were effectively inhibited by the four different concentrations of the samples (1.0 mg/mL, 0.1 mg/mL, 0.01 mg/mL, and 0.001 mg/mL). According to the data in Supplementary Table S8, Resveratrol exhibited the highest antioxidant activity across all concentrations, particularly at 1 mg/mL, where it achieved 92% inhibition of ABTS radicals. The root extract followed closely, demonstrating 88% inhibition at the same concentration. Rutin also displayed strong antioxidant potential, with 78% inhibition at 1 mg/mL. However, its activity diminished more rapidly at lower concentrations compared to resveratrol and roots. In comparison, the extracts obtained from the aerial parts were only moderately active, achieving 37% inhibition at 1 mg/mL concentration and this activity decreased at lower concentrations. Astragalin demonstrated the weakest antioxidant activity, achieving only 25% inhibition at 1 mg/mL, and showing minimal effectiveness at lower concentrations.

**FIGURE 4:**
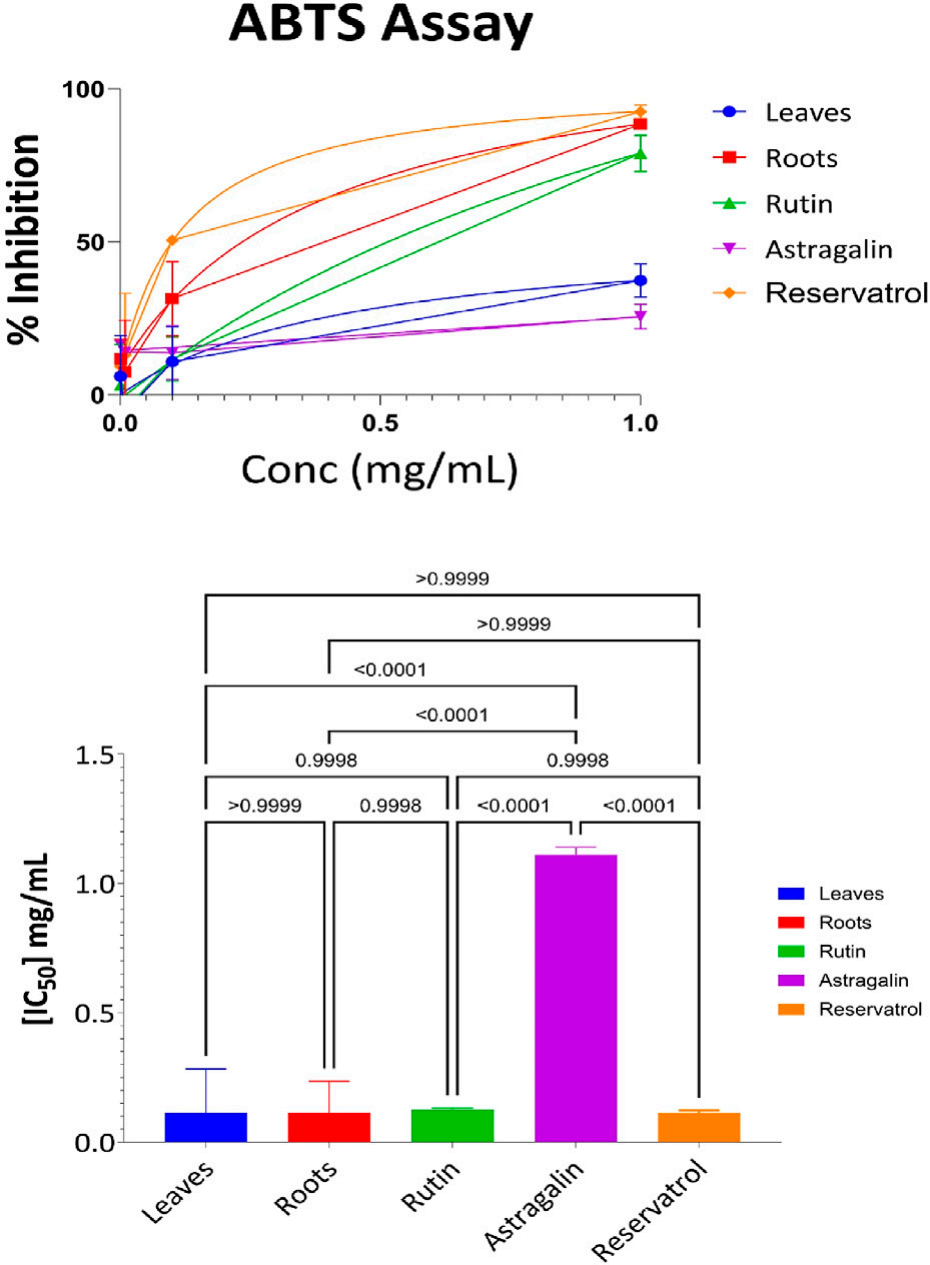
ABTS antioxidant activity: percent inhibition and IC_50_ with statistical comparisons (One-way ANOVA and Tukey’s test) data are expressed as mean +- SD and n = 3.

The results from the ABTS assay, as visualized in Figure 4, provide further insights into the antioxidant capacity of the samples. The ABTS radical scavenging assay was performed to evaluate the antioxidant potential of the tested samples. The IC_50_ values revealed that Resveratrol (IC_50_ = 0.1 mg/mL) exhibited the highest antioxidant activity, followed closely by Roots (IC_50_ = 0.11 mg/mL), Leaves (IC_50_ = 0.12 mg/mL), and Rutin (IC_50_ = 0.125 mg/mL), where Astragalin displayed the weakest antioxidant activity (IC_50_ = 1.1 mg/mL). Statistical analysis using one-way ANOVA and Tukey’s multiple comparison test showed no significant differences between Leaves, Roots, Rutin, and Resveratrol (p > 0.9999), indicating similar antioxidant potential among these samples. However, Astragalin exhibited significantly lower antioxidant activity compared to all other tested compounds (p < 0.0001), confirming its weaker radical scavenging capacity. These findings reinforce the strong antioxidant potential of Leaves, Roots, Rutin, and Resveratrol, with Astragalin demonstrating a markedly lower ability to neutralize ABTS radicals.

#### 3.3.3 FRAP result of the following fraction aerial part, root, resveratrol, astragalin, and rutin

**FIGURE 5:**
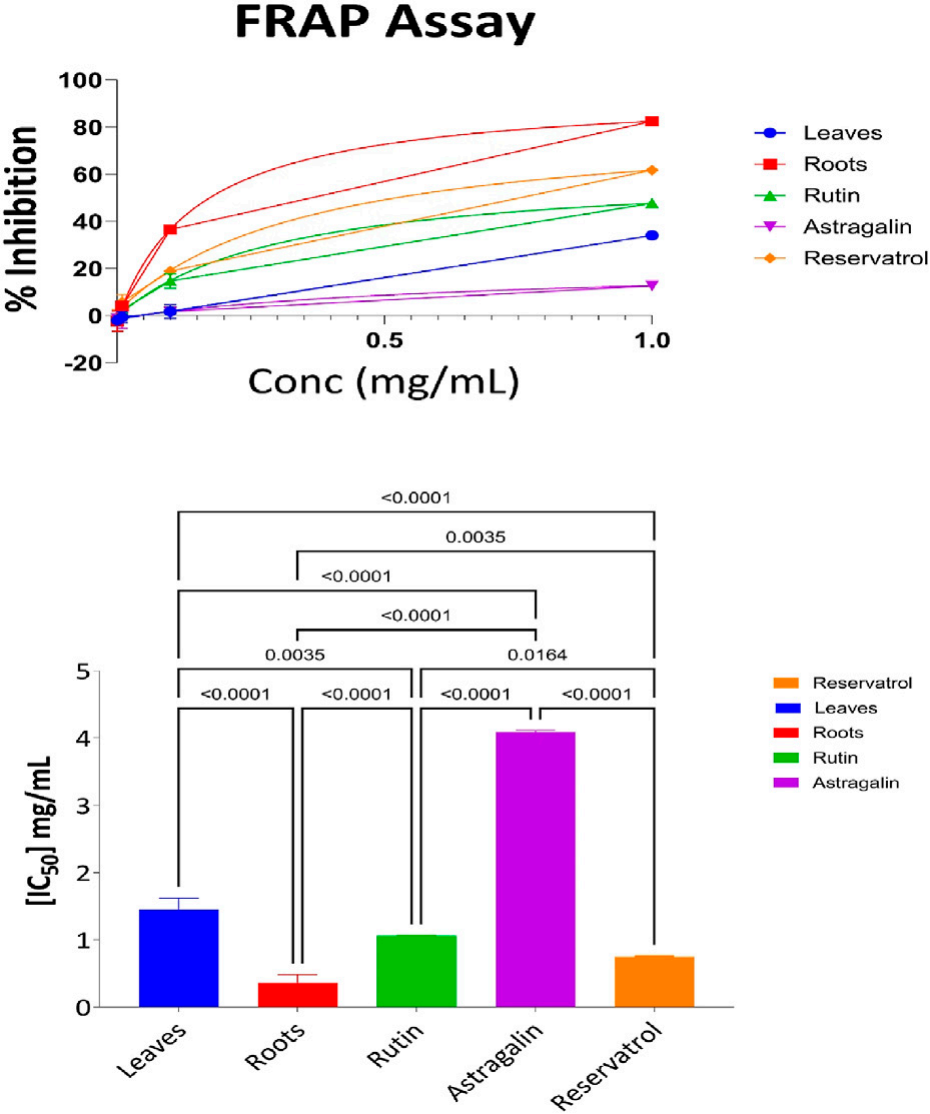
FRAP antioxidant activity: percent inhibition and IC_50_ with statistical comparisons (One-Way ANOVA and Tukey’s test) data are expressed as mean +- SD and n = 3.

The Ferric Reducing Antioxidant Power (FRAP) assay quantified the antioxidant potential of the aerial parts, roots, resveratrol, astragalin, and rutin. This assay evaluates the activity of samples in the reduction of ferric ions (Fe^3+^) to ferrous ions (Fe^2+^) as a defined measure of antioxidant activity. Supplementary Table S9 shows the percentage of ferric ion reduction at various concentrations for each of the samples. At the highest concentration of 1 mg/mL, the root extract was found to have the highest level of antioxidant activity with 82% ferric ion reduction. This was followed by resveratrol at 61% and Rutin at 47% respectively. The aerial parts exhibited moderate antioxidant activity with 34% ferric ion reduction, while Astragalin had the least activity reducing 12% of the ferric ions. At lower concentrations such as (0.01 mg/mL and 0.001 mg/mL), the samples in general showed a significant reduction in antioxidant activities, with some being negative values, showing a lack of or very little activity. This highlights the importance of concentration in the investigation of antioxidant screening by FRAP where high amounts of the materials are essential to produce enhanced antioxidant activities. The results presented in Figure 5 show IC_50_ of aerial parts, roots, Resveratrol, Astragalin, and Rutin as a free radical scavenging effect. The Ferric Reducing Antioxidant Power (FRAP) assay was performed to assess the reducing capacity of the tested samples. The IC_50_ values indicated that Roots (IC_50_ = 0.36 mg/mL) exhibited the highest reducing power, followed by Resveratrol (IC_50_ = 0.7 mg/mL), Rutin (IC_50_ = 1.06 mg/mL), and Leaves (IC_50_ = 1.4 mg/mL), Astragalin showed the weakest reducing capacity (IC_50_ = 4.08 mg/mL). Statistical analysis using one-way ANOVA and Tukey’s multiple comparison tests confirmed significant differences (p < 0.0001) between Leaves and Roots, as well as between Astragalin and all other tested samples, indicating substantial variations in antioxidant potential. Additionally, significant differences were observed between Rutin and Resveratrol (p = 0.0164) and between Roots and Resveratrol (p = 0.0035), highlighting their distinct reducing capacities. These results suggest that Roots possess the strongest ferric reducing power, followed by Resveratrol, while Astragalin exhibited significantly lower antioxidant activity compared to all other samples.

#### 3.3.4 CUPRAC antioxidant results for aerial part, root, resveratrol, astragalin, and rutin

The evaluation of the antioxidant activities of different fractions of the aerial part, root, resveratrol, astragalin, and rutin was carried out using the Cupric Reducing Antioxidant Capacity (CUPRAC) assay. This assay evaluates the ability of antioxidant compounds to reduce the cupric ions (Cu^2^⁺) to the cuprous ions (Cu⁺) which gives a good measure of its antioxidant activities. Supplementary Table S10 presents the percentage of reduction at various concentrations for each sample. At the maximum concentration (1 mg/mL), the antioxidant activity was the most substantial as 95% of the root extract was reduced with the decreasing order of activity following 94% of rutin and 93% of resveratrol. Astragalin could also display potent antioxidant effects with only an 85% reduction, whereas the aerial parts exhibited moderate activity with a 68% reduction. Decreased values of antioxidant activities were observed in samples at lower concentrations with the negative reduction values occurring at 0.01 mg/mL, and 0.001 mg/mL suggesting no activity or negligible at those concentrations.

**FIGURE 6:**
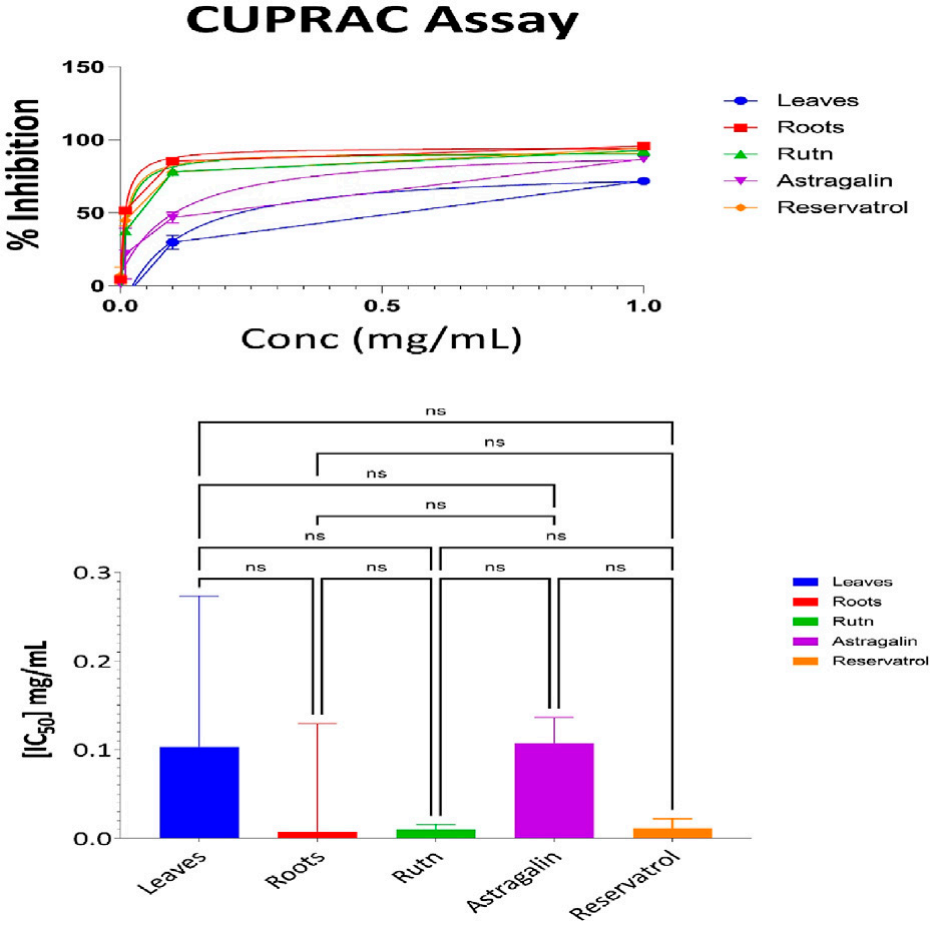
CUPRAC antioxidant activity: percent inhibition and IC_50_ with statistical comparisons (One-way ANOVA and Tukey’s Test) data are expressed as mean +- SD and n = 3.

The IC_50_ values, which represent the concentration required to achieve 50% inhibition, provide a more detailed comparison of the antioxidant efficacy of the samples. Figure 6 illustrates the IC_50_ values for the aerial parts, roots, resveratrol, astragalin, and rutin. The CUPRAC assay results indicate the antioxidant capacities of Leaves, Roots, Ruth, Astragalin, and Resveratrol, evaluated through % inhibition at concentrations of 0.001, 0.01, 0.01 and 1.0 mg/mL. All samples showed no statistically significant differences (marked as “ns”) in % inhibition across the tested concentrations, suggesting comparable short-range antioxidant effects at these specific doses. The CUPRAC assay was conducted to evaluate the antioxidant capacity of the tested samples. The IC_50_ values revealed that Roots (IC_50_ = 0.06 mg/mL) exhibited the strongest antioxidant activity, followed by Rutin (IC_50_ = 0.09 mg/mL) and Resveratrol (IC_50_ = 0.12 mg/mL). Astragalin (IC_50_ = 0.126 mg/mL) demonstrated slightly weaker activity, while Leaves (IC_50_ = 0.7 mg/mL) showed the lowest antioxidant potential among the tested samples. These findings highlight the superior antioxidant activity of Roots, likely due to the presence of highly effective reducing agents, as measured by the CUPRAC method.

Statistical analysis using one-way ANOVA revealed no significant differences in antioxidant activity between Roots, Rutin, Resveratrol, and Astragalin (p > 0.05).

#### 3.3.5 Statistical analysis and correlation between antioxidant activity measuring methods

**FIGURE 7:**
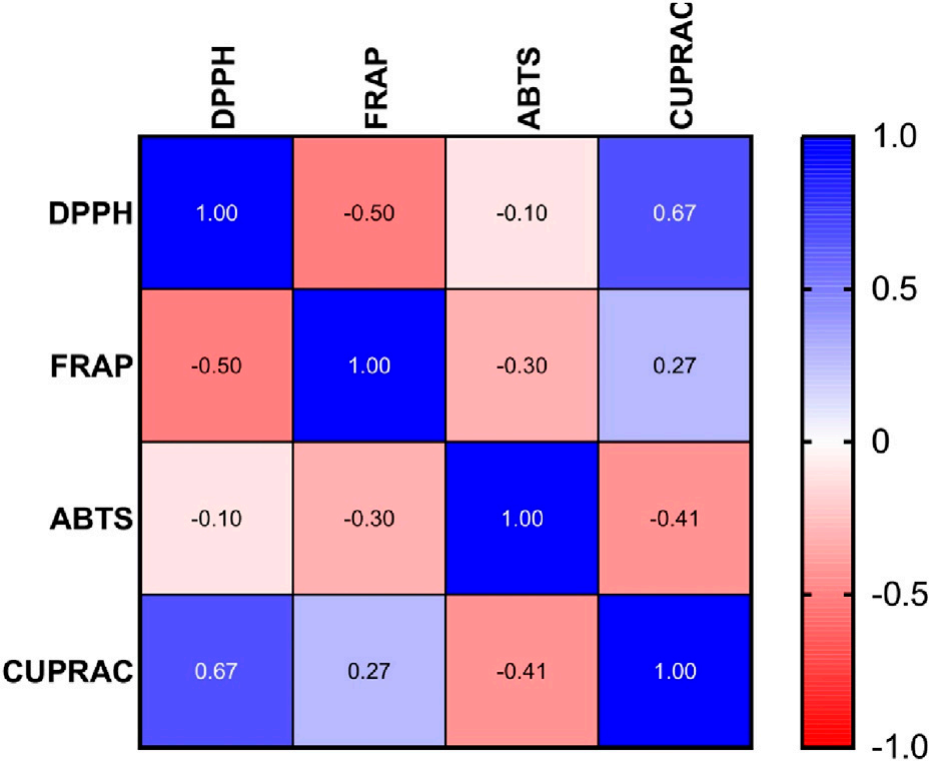
Pearson correlation coefficient analysis of IC_50_ values in DPPH, FRAP, ABTS, and CUPRAC antioxidant assays.

Interpretation of Pearson Correlation Coefficients in the Context of Antioxidant Assays, A comparative analysis of the antioxidant potential among the samples is illustrated in Figure 7.

The Pearson correlation matrix in the provided figure represents the relationships between four used antioxidant assays DPPH, FRAP, ABTS and CUPRAC.

Each of these assays measures antioxidant potential but through different mechanisms. Correlations among them indicate similarities or differences in how antioxidants behave in different experimental conditions.

Correlations among them indicate similarities or differences in how antioxidants behave in different experimental conditions.

1. Strong Positive Correlation: DPPH vs. CUPRAC (r = 0.67)

The IC50 value for DPPH and CUPRAC shows strong linear correlation, suggesting that samples have high activity in DPPH also will have high activity in CUPRAC.

2. Moderate Negative Correlation: DPPH vs. FRAP (r = −0.50)

A moderate inverse relationship indicates that samples with lower IC_50_ values in DPPH (higher antioxidant activity) tend to have higher IC_50_ values in FRAP meaning lower antioxidant activity 3. Weak Negative Correlation: DPPH vs ABTS (r = −0.10)

The weak negative correlation suggests minimal relationship between these assays in terms of IC_50_ values.

4. Weak Positive Correlation: FRAP vs CUPRAC (r = 0.27)

A weak but positive association exists between these two assays, indicating that some trends in antioxidant activity are shared.

5. Moderate Negative Correlation: ABTS vs. CUPRAC (r = −0.41)

A moderate inverse correlation implies that high antioxidant activity in ABTS (low IC_50_) is moderately associated with lower antioxidant activity in CUPRAC.

6. Weak Negative Correlation: FRAP vs. ABTS (r = −0.30)

A weak inverse correlation suggests that there is little overlap in the antioxidant mechanisms measured by FRAP and ABTS.

### 3.4 MTT assay results and statistical analysis

**FIGURE 8:**
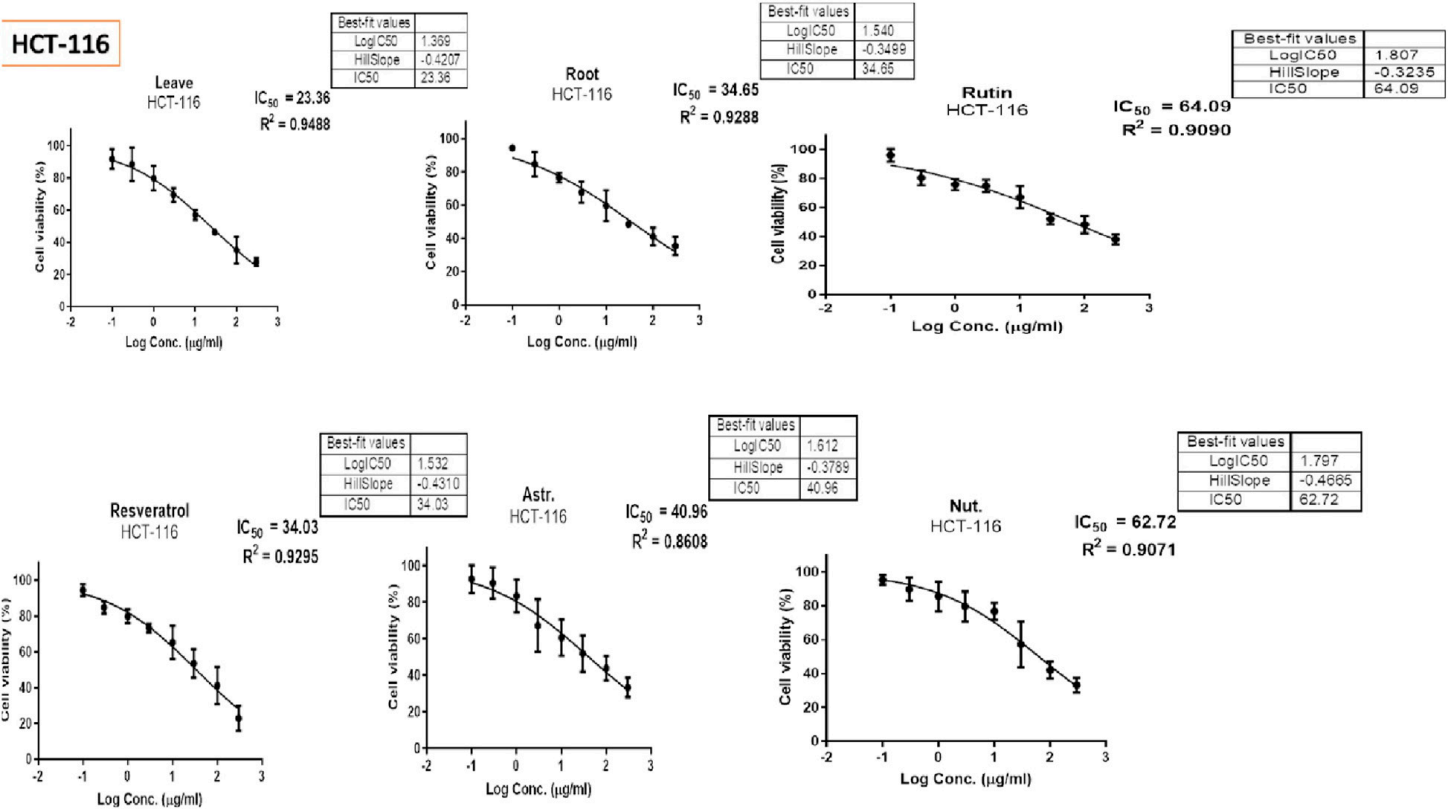
Antiproliferative activity of various extracts and compounds against HCT-116 cells.

**FIGURE 9:**
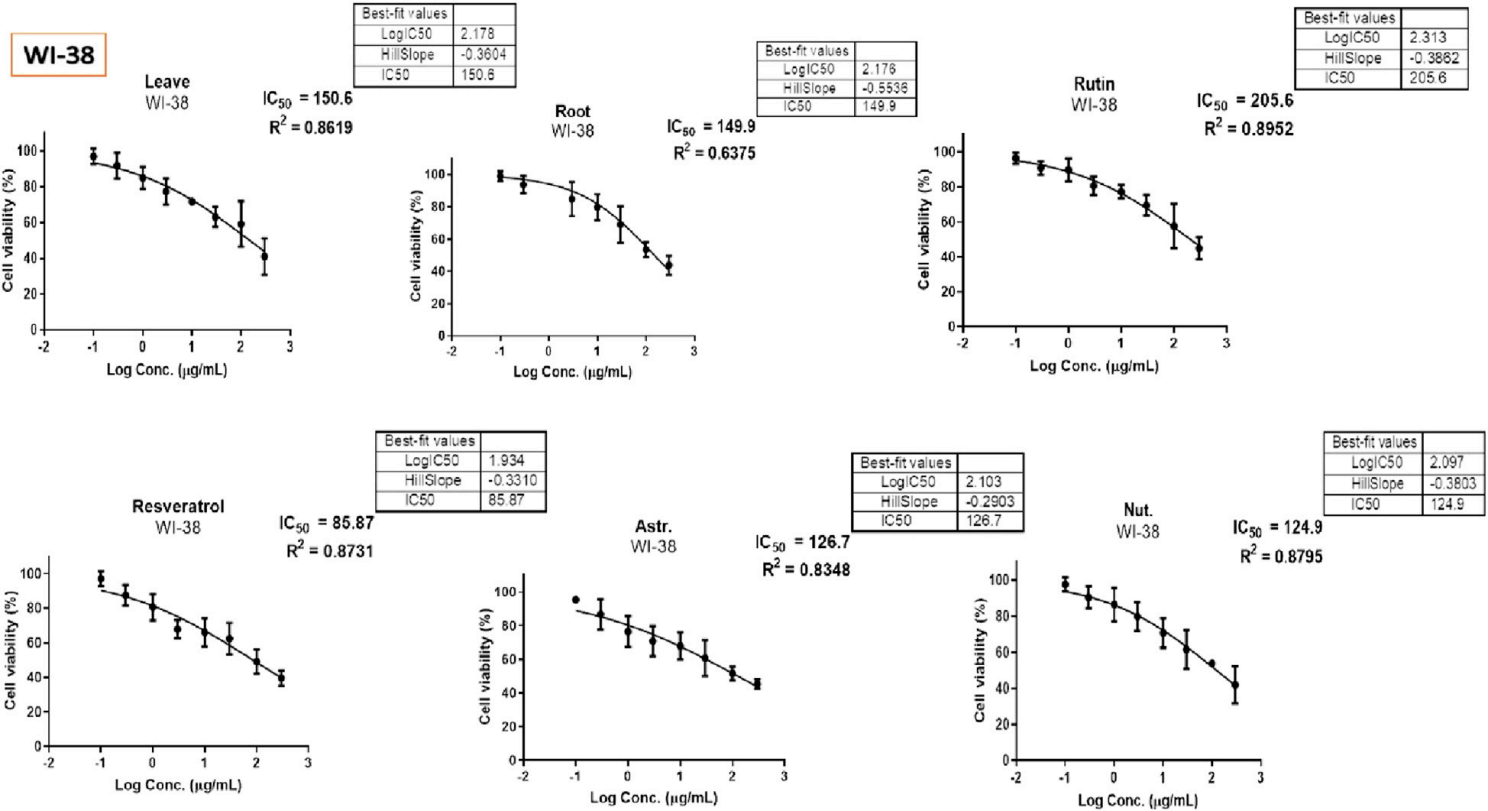
Antiproliferative activity of various extracts and compounds against WI-38 normal cells.

The MTT assay was accomplished to assess the cell’s viability in response to treatments with different extracts and compounds and the result is explained in Table 2, which shows the percent of antiproliferation activity against the cancer cell human colon (HCT-116) (Figure 8 and effect against the normal lung fibroblast (WI-38) (Figure 9) cells to show the safety of those extracts and compounds, all cells treated for 72 h.

**TABLE 2:** Antiproliferative activity of compounds on HCT-116 and WI-38 cell lines.

Comp	Sample	*In vitro* cytotoxicity [IC_50_, µg/mL ± SD]^a^	
		HCT-116	WI-38
1	leave	23.26 ± 2.30	150.60 ± 2.90
2	Roots	34.65 ± 2.17	149.90 ± 4.75
3	Rutin	64.09 ± 2.06	205.60 ± 2.53
4	Astragalin	40.96 ± 2.23	126.70 ± 2.87
5	Resveratrol	34.03 ± 2.45	85.87 ± 2.74
Nutlin		62.72 ± 3.15	124.90 ± 3.06

**FIGURE 10:**
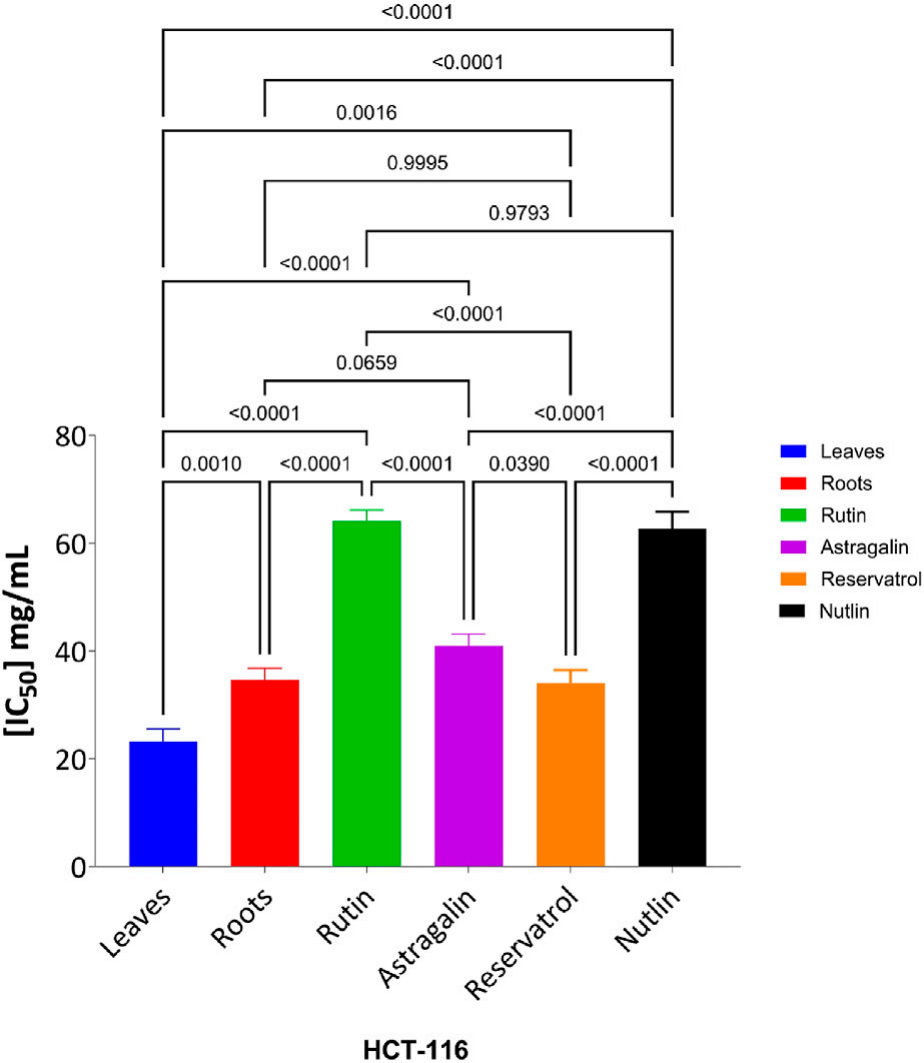
Effect of tested compounds on HCT-116 cell viability: one-way ANOVA and Tukey’s post-hoc analysis.

**FIGURE 11:**
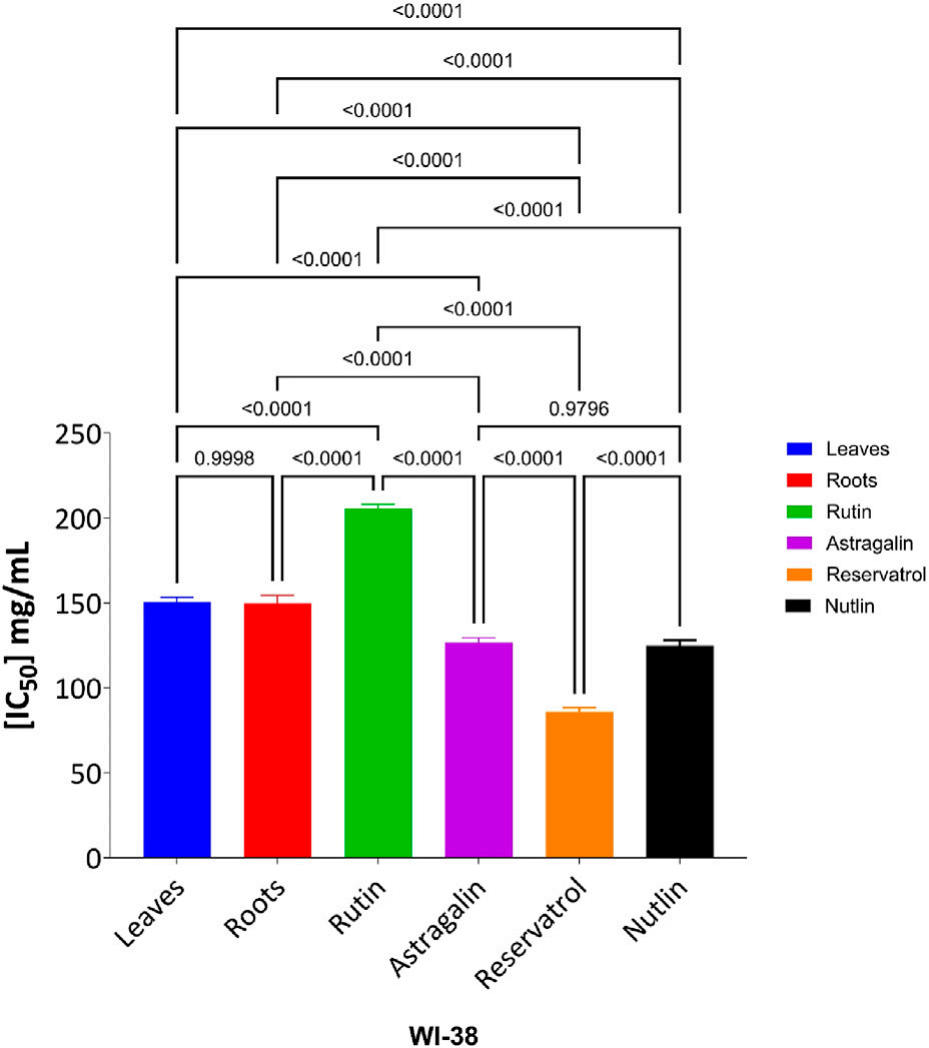
Effect of tested compounds on WI-38 cell viability: one-way ANOVA and Tukey’s post-hoc analysis.

The cytotoxic effects of the tested compounds on HCT-116 and WI-38 cells were statistically analyzed. Significant differences among the tested samples were determined, and *post hoc* comparisons were conducted to identify group variations as shown in Figure 10 which shows the result on HCT-116 and Figure 11 explains the statistical result on the WI-38 cell line.

The MTT assay results, analyzed using one-way ANOVA and Tukey’s multiple comparison tests, provide insights into the cytotoxic effects and selectivity of the tested compounds on HCT-116 colorectal cancer cells and WI-38 normal cells (Khayat et al., 2022). The analysis compared the cytotoxic activity of five natural compounds (Leaves, Roots, Rutin, Astragalin, and Resveratrol) with Nutlin, a well-established MDM2 inhibitor used as a reference compound (Basha and Mohan, 2024). In HCT-116 cells, Nutlin exhibited an IC_50_ of 62.72 ± 3.15 μg/mL, serving as the benchmark for cytotoxicity. Among the tested compounds, Leaves demonstrated the highest cytotoxicity (IC_50_ = 23.26 ± 2.30 μg/mL, p < 0.0001 vs. Nutlin), followed by Roots (IC_50_ = 34.65 ± 2.17 μg/mL, p < 0.0001 vs. Nutlin), Resveratrol (IC_50_ = 34.03 ± 2.45 μg/mL, p < 0.0001 vs. Nutlin), and Astragalin (IC_50_ = 40.96 ± 2.23 μg/mL, p < 0.0001 vs. Nutlin), whereas Rutin (IC_50_ = 64.09 ± 2.06 μg/mL, p = 0.979 vs. Nutlin) exhibited comparable cytotoxicity to the reference compound. These findings suggest that Leaves, Roots, Resveratrol, and Astragalin possess stronger anticancer activity than Nutlin, indicating the potential anticancer activity for those extracts and compounds.

In contrast, cytotoxicity evaluation in WI-38 normal cells revealed significantly different trends, emphasizing compound selectivity and potential therapeutic windows. Leaves and Roots exhibited high IC_50_ values (less toxicity) in WI-38 cells compared to HCT-116, indicating selective cytotoxicity toward cancer cells. Tukey’s multiple comparison tests showed no significant difference between Leaves and Roots in normal cells (p = 0.9998), suggesting a similar safety profile. However, all other comparisons (Leaves vs. Rutin, Astragalin, Resveratrol, and Nutlin; p < 0.0001) indicated significant differences, highlighting the lower toxicity of Leaves and Roots in normal cells. Moreover, Rutin and Resveratrol exhibited significantly higher toxicity in WI-38 cells than in Leaves and Roots (p < 0.0001), suggesting a potential risk of non-selective cytotoxicity. Astragalin, while cytotoxic in cancer cells, showed a non-significant difference compared to Nutlin in normal cells (p = 0.9796), indicating a safety profile similar to the reference drug. Overall, these findings suggest that Leaves and Roots exhibit the best selectivity between cancer and normal cells, making them promising candidates for further anticancer investigations.

## 4 Discussion

The results from the phytochemical screening of the aerial part extracts indicated that there exist several classes of important metabolites such as; flavonoids and coumarins, phenol derivatives, terpenes, and cardiac glycosides as shown by previous studies (Annaz et al., 2022). Similar findings have been reported in previous studies, where Capparis spinosa was recognized for its rich flavonoid and polyphenolic content, contributing to its therapeutic properties (Castillo et al., 2024).

### 4.1 Antioxidant activity

The five samples that were subjected to four standard antioxidant assays indicated Rutin, Roots, Resveratrol, Leaves, and Astragalin were also measured for their IC_50_ values and antioxidant potential in these assays-DPPH, FRAP, ABTS, and CUPRAC. From these tests, all the samples were assessed for their scavenging activities and reductive capabilities, and differences were observed in the Levels of Antioxidant activity in different methods used.

The DPPH method is an established approach for evaluating the free radical scavenging activity (Gulcin and Alwasel, 2023). In this assay, Rutin emerged as the potent antioxidant with the least IC_50_ having values of 0.013 mg/mL. This shows that the compound can scavenge free radicals at remarkably low concentrations; our results align with these findings (Brighente et al., 2007; Wang et al., 2021; Zhou et al., 2023). The root extract followed closely with the IC_50_ concentrations of 0.017 mg/mL, which shows that this also has a good free radical scavenging ability. Resveratrol on the other hand, although the IC_50_ value is just a bit more than for Rutin, showing 0.032 mg/mL, exhibited high efficiency in this assay as shown by other study (Yang et al., 2008). Leaves and Astragalin had much higher IC_50_ values thus implying a lower capacity to exert antioxidant effects. A particularly high IC_50_ value of astragalin which was 1.2 mg/mL; it is the least effective against DPPH radicals.

In the ABTS assay Resveratrol exhibited the highest activity with an IC_50_ value of 0.1 mg/mL that making him a potent free radical scavenging compound as approved by a previous study (Rosiak et al., 2022). This was closely followed by the Roots (0.11 mg/mL) and Leaves (0.121 mg/mL) suggesting that these samples are reasonably efficient in scavenging ABTS radicals as shown by other studies (Annaz et al., 2022). Though Astragalin did not possess ABTS scavenging activity and had a slightly higher IC_50_ value of 1.1 mg/mL confirming its consistently lower antioxidant capacity across different assays as also approved by other studies (Li et al., 2017). Rutin on the other hand also has an IC50 value close to that of roots 0.125 suggesting its high activity in ABTS assay as demonstrated by this study (Devkota et al., 2023).

In the FRAP assay which assays the ability of the samples to convert Fe3+ to Fe2+ (Mishra et al., 2022), it was observed that the roots extract had the greatest activity with an IC_50_ value of 0.36 mg/mL (Rajhi et al., 2021). This implies that there exist compounds present in the roots that enhance the effectiveness of this reduction most, rendering it the strongest in terms of ferrous reducing capacity. On the other hand, leaf extracts, with an IC_50_ of 1.4 mg/mL, showed low reducing capacity, while Rutin in the FRAP assay showed low activity with an IC50 value of 1.06 mg/mL. Resveratrol demonstrates high activity just a second after the roots extract with IC50 0.7 mg/lm aligns with another study showing also a high activity for it in FRAP assay (Rajhi et al., 2021). The high IC_50_ value of 4.0 mg/mL for Astragalin was indicative of low antioxidant activity, which was due to a lower ability to engage in redox reactions relative to the other samples (Li et al., 2017). In CUPRAC activity test The most active out of all the tested samples turned out to be Root extract with the IC_50_ of 0.06 mg/mL as approved by Zamani et al. (2023), Followed by Rutin with IC_50_ of 0.09 mg/mL rendering them the second potent one in CUPRAC assay. This result brings out Roots cupric-reducing activity at the maximum level (Zamani et al., 2023). Resveratrol on the other hand showed also high antioxidant activities IC_50_ values of 0.012 mg/m (Zamani et al., 2023)L. In this case, the value of IC_50_ for the Leaves is higher at 0.7 mg/mL, which suggests the presence of lesser antioxidant activity despite the majority of studies showing high activity for *Capparis spinosa* leaves extract (Beldi et al., 2022). These observations reinforce the necessity of additional statistical measurements to characterize and compare any antioxidant action.

The cytotoxicity study of the two extracts (leaves and roots) of *C. spinosa* along with the three natural compounds rutin, astragalin and resveratrol were analyzed using one-way ANOVA and Tukey’s multiple comparison test to provide insight into the anticancer activity and selectivity of the tested compounds (Potočnjak et al., 2022). The result revealed that the leaves and root extracts showed highest toxicity against the HCT-116 cancer cells and the lower against normal cells WI-38 normal cells. The selectivity is crucial, as an ideal therapeutic agent should effectively target cancer cells while sparing normal cells (Garg et al., 2024).

In this study, Nutlin was used as a reference compound and showed IC₅₀ of 62.72 ± 3.15 μg/mL in HCT-116 cells, consistent with its role in a previous study in activity and restoring the P53 role as an anticancer agent (Kumari et al., 2022). However several of the compounds included in this study particularly the leaves and roots exhibited significantly higher activity with Leaves (IC_50_ = 23.26 ± 2.30 μg/mL) and Roots (IC_50_ = 34.65 ± 2.17 μg/mL), demonstrated significantly higher cytotoxicity than Nutlin (p < 0.0001) suggesting their potential activity as anticancer agent this results along with previous similar study like activity of leaves on HCT-116 (Abdul Samad, 2024; El-Berawey, 2024). The significant cytotoxicity of Resveratrol (IC_50_ = 34.03 ± 2.45 μg/mL, p < 0.0001 vs. Nutlin) aligns with prior findings on its ability to induce apoptosis, inhibit proliferation, and enhance oxidative stress in colorectal cancer cells (Gündoğdu and Özyurt, 2023). The differential cytotoxicity observed between HCT-116 and WI-38 cells is a crucial finding, as Leaves and Roots extracts displayed significantly higher IC_50_ values in WI-38 cells, indicating lower toxicity in normal cells. This selectivity suggests a potential therapeutic advantage, as compounds with a high cancer-to-normal cell cytotoxicity ratio are preferred in drug development to minimize systemic toxicity (Ramos et al., 2021). Additionally, Rutin (Ur Rahman and Panichayupakaranant, 2025) and Resveratrol (El Omari et al., 2021) exhibited relatively little higher toxicity in WI-38 cells but still kept their safety profile against normal cells and selectivity against HCT-116. Astragalin, which showed strong cytotoxicity against cancer cells (Chen et al., 2017), exhibited a non-significant difference compared to Nutlin in normal cells (p = 0.9796), suggesting a comparable safety profile to the reference MDM2 inhibitor.

The observed cytotoxic effects can be attributed to multiple mechanisms, including MDM2 inhibition, apoptosis induction, and oxidative stress modulation, which are well-documented anticancer pathways (Khan et al., 2020; Jazvinšćak Jembrek et al., 2021; Yarmohammadi et al., 2021). Our study provides a strong foundation for the further development of selective anticancer agents. Future investigations should focus on detailed mechanistic studies, including apoptosis assays, cell cycle analysis, and gene expression profiling of MDM2 and related pathways to fully elucidate the potential of these compounds.

## 5 Conclusion

The current study highlights the importance of performing multiple assays to assess antioxidant capacity, given the variability observed across different tests, such as DPPH, FRAP, ABTS, and CUPRAC. Among the tested samples, certain compounds, particularly Rutin, displayed strong antioxidant potential, suggesting its role in radical scavenging and reducing power. The root extract also demonstrated notable antioxidant activity. While Resveratrol and the aerial parts showed moderate activity, Astragalin exhibited minimal antioxidant capability across assays. Rutin and root extracts appear promising for further exploration as natural antioxidants, while Resveratrol may also hold potential. Additionally, the MTT assay results revealed that certain samples exhibit varying degrees of cytotoxicity against HCT-116 cancer cells, with leaf extracts showing potent and selective anticancer activity. The root extract and Resveratrol also demonstrated moderate cytotoxicity, although with slightly less selectivity. These findings suggest that the cytotoxic potential, coupled with selectivity, is crucial in identifying viable anticancer agents. Further research is necessary to elucidate the mechanisms underlying these activities and to optimize their therapeutic potential, aiming to balance efficacy with minimal toxicity to normal cells.

## Data Availability

The raw data supporting the conclusions of this article will be made available by the authors, without undue reservation.
