# Supplementary material for: Antioxidant activity and selective cytotoxicity in HCT-116 and WI-38 cell lines of LC-MS/MS profiled extract from Capparis spinosa L
Source: Front Chem. 2025 Apr 10;13:1540174. doi: 10.3389/fchem.2025.1540174 (PMC12018432; doi:10.3389/fchem.2025.1540174)
Supplement: Supplementary file 1 [file DataSheet1.pdf]

**Supporting Information**

**Antioxidant Activity, and Selective Cytotoxicity in HCT-116 and WI-38 cell Lines of LC-MS/MS profiled extract from *Capparis spinosa* L.**

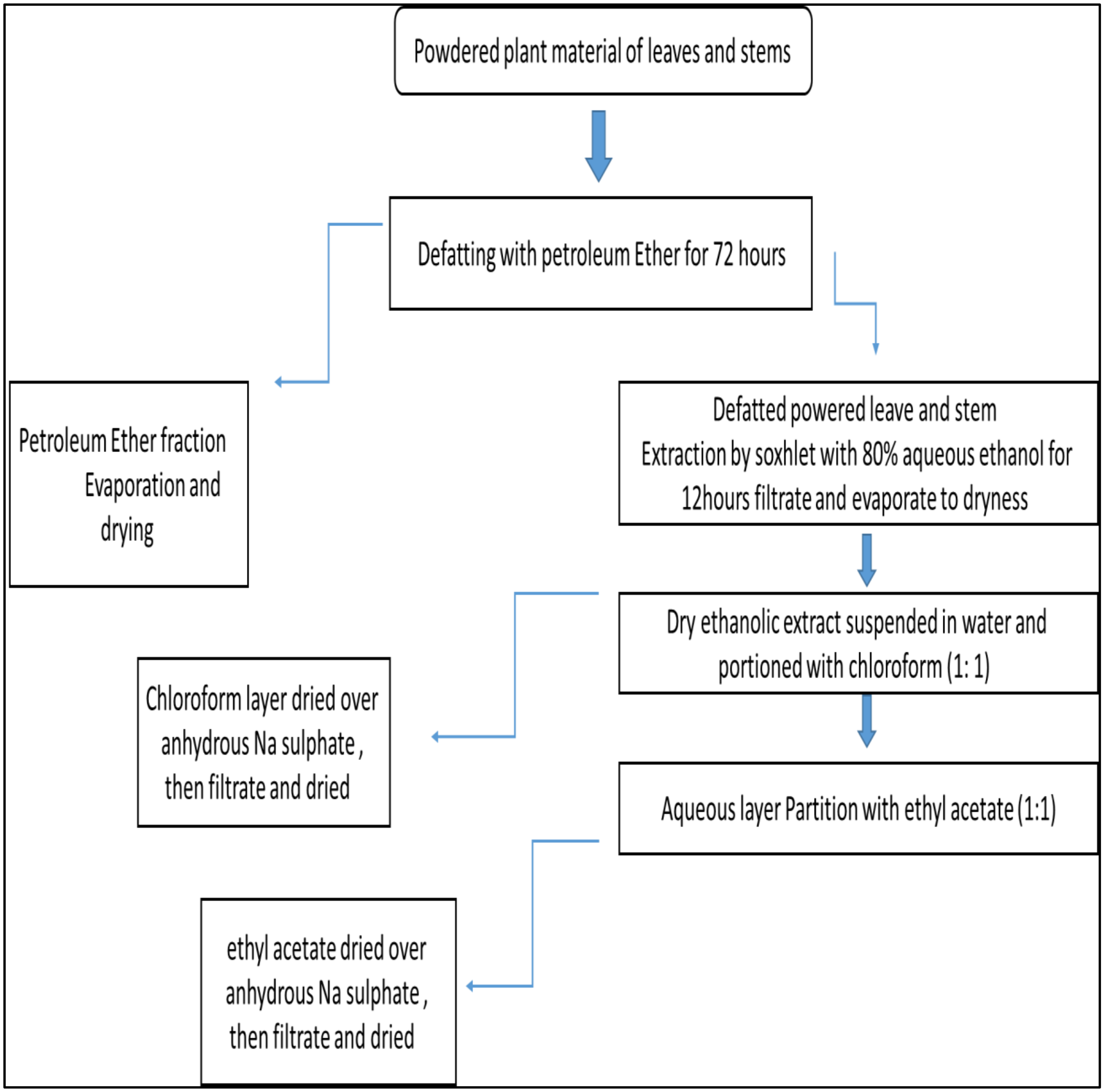


**Figure s1:** Schematic diagram for fractionation of leaves and stem of *Capparis spinosa* crude extracts

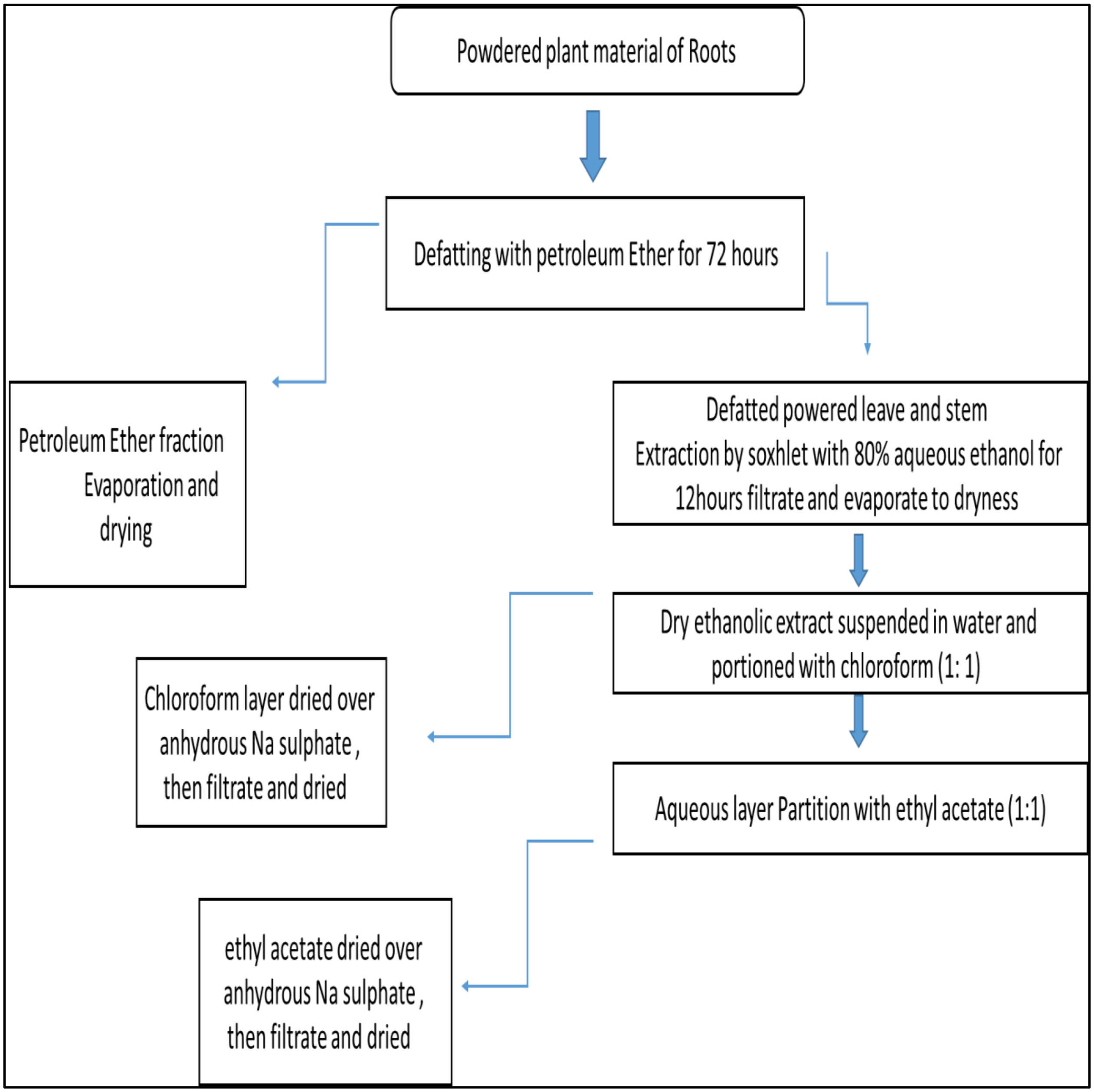


**Figure s2:** Schematic diagram for fractionation of roots of *Caparis spinosa* crude extracts

## LC-MSMS result of ethyl acetate fractions in aerial parts and root

**Table s1**. LC-MS/MS reported compounds in aerial parts of *C. spinosa* ethyl acetate fraction

<table>
<tr><th>Compound</th><th>Mw</th><th>RT</th><th>Mass fragmentation report</th><th>Structure</th></tr>
<tr><td>Apigenin 7-glucoside</td><td>433</td><td>7.2</td><td>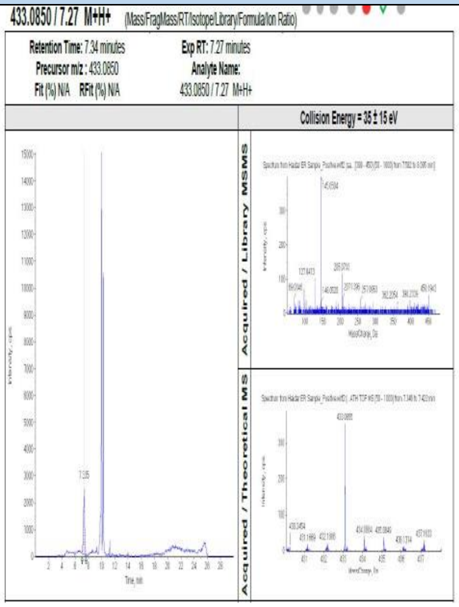
</td><td>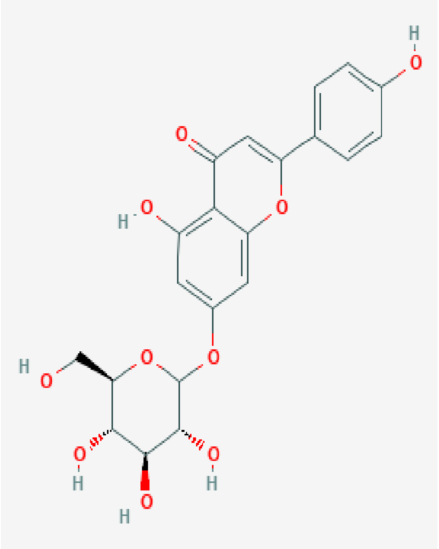</td></tr>
<tr><td>Kaempferol</td><td>287</td><td>12.19</td><td>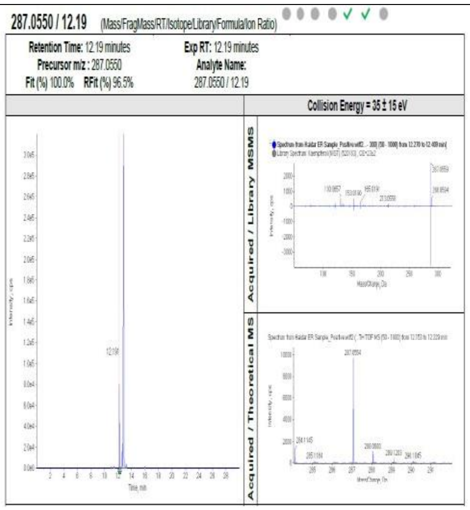
</td><td>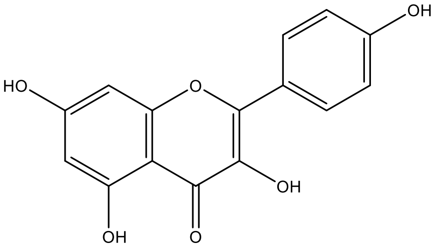

Kaempferol</td></tr>
</table>

| | | | | |
|---|---|---|---|---|
| Rutin | 644 | 12.4 | 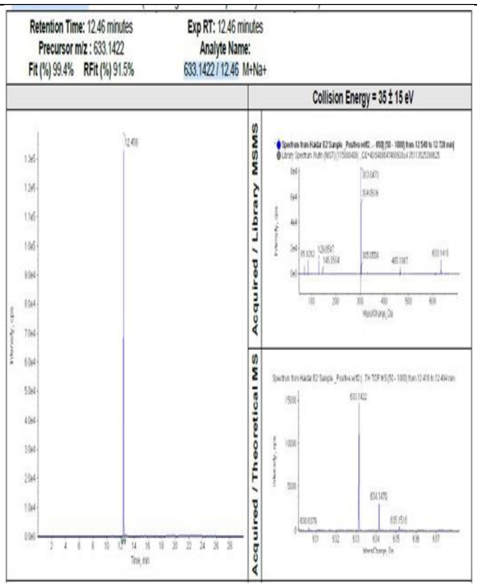 | 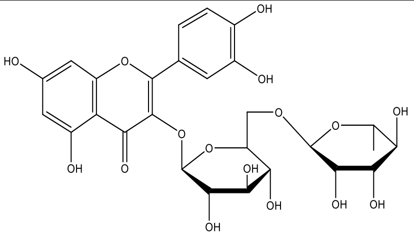 Rutin |
| Astragalin | 449 | 10.6 | 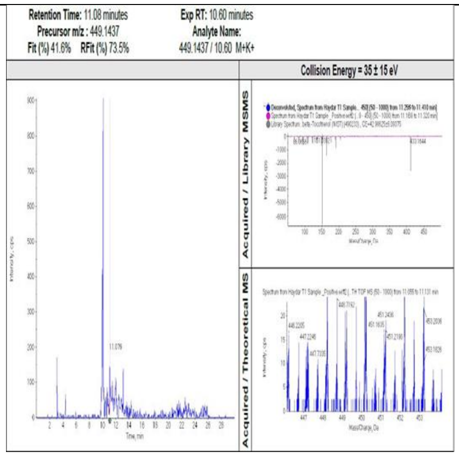 | 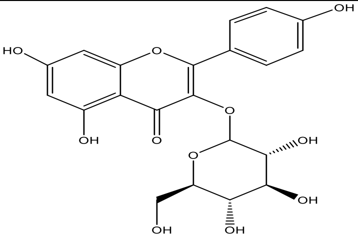 Astragalin |
| Isorahmnetine | 316 | 12.04 | 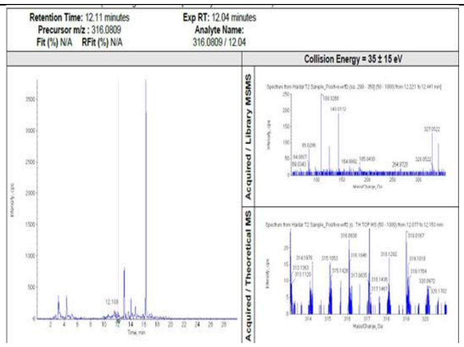 | 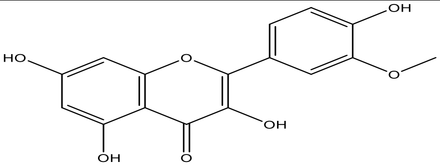 isorhamnetine |

| | | | | |
|---|---|---|---|---|
| Cumaric acid | 165 | 5.6 | 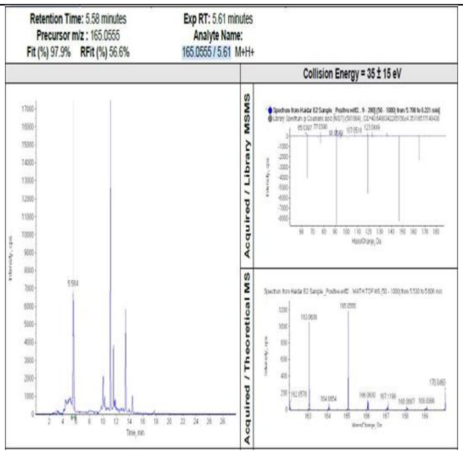 | 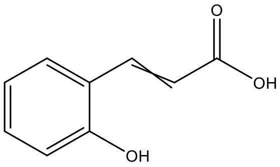 2-Hydroxycinnamic acid |
| 2,5-dimethoxyflavon | 282 | 6 | 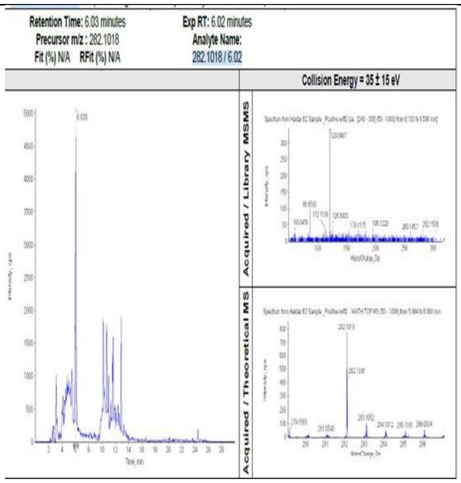 | 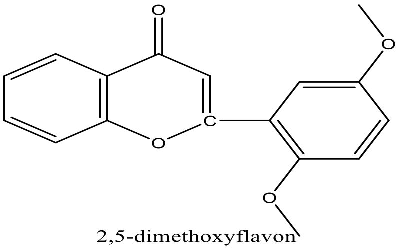 2,5-dimethoxyflavon |
| Quercetin | 302 | 11.7 | 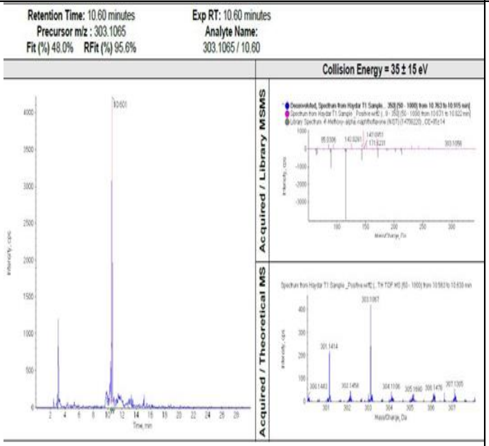 | 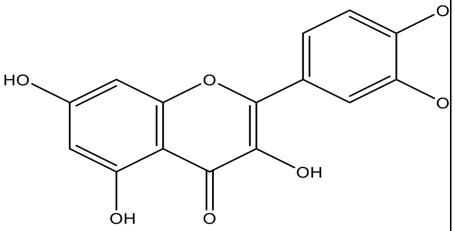 Quercetin |

| | | | | |
|---|---|---|---|---|
| Cyanidin | 287 | 8.8 | 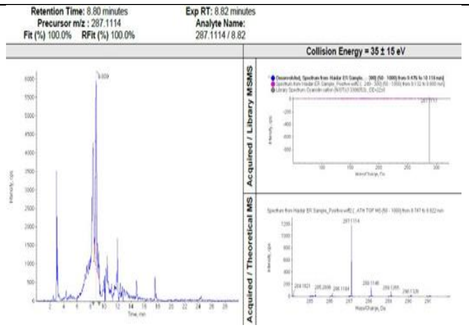 | 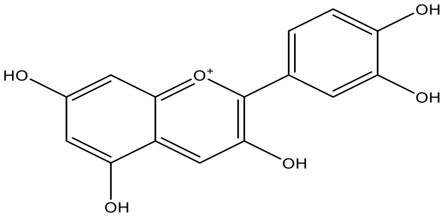<br>Cyanidin |
| Resveratrol | 270 | 14.9 | 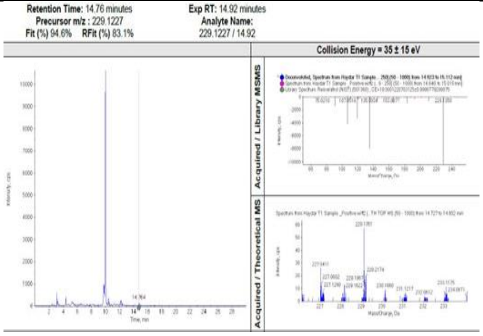 | 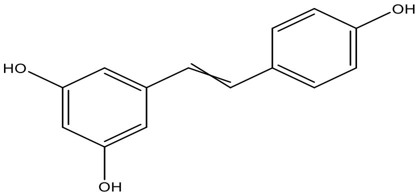<br>Resveratrol |

**Table s2.** LC-MS reported compounds in Roots of C. *spinosa* ethyl acetate fraction

| Compound | Mw | RT | Mass spectra | Structure |
|---|---|---|---|---|
| Nicotinic acid | 124 | 4.3 | 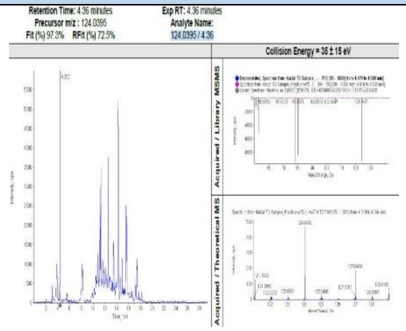 | 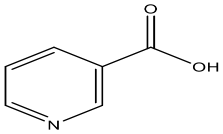<br>Nicotinic acid |
| Cyano-7-ethoxycoumarin | 216 | 9.9 | 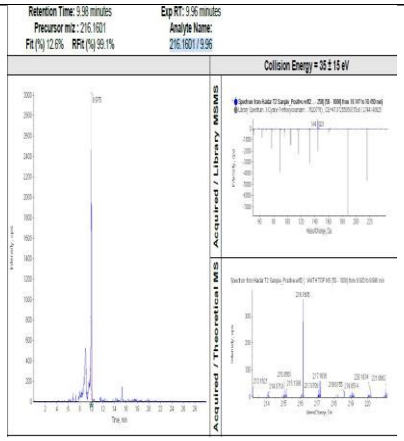 | 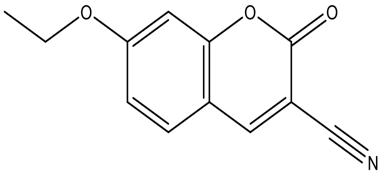<br>Cyano-7-ethoxycoumarin |

| | | | | |
|---|---|---|---|---|
| 3-Hydroxy-7,8,2',3'-tetramethoxyflavone | 359 | 13.9 | 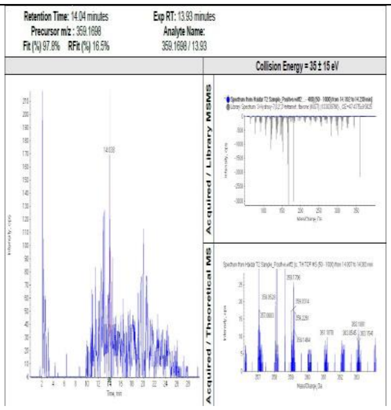  | 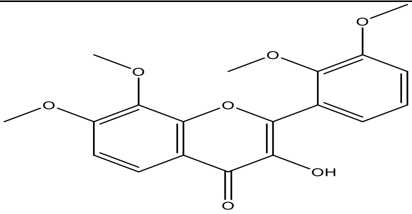  3-Hydroxy-7,8,2',3'-tetramethoxyflavone |
| Quinone | 109 | 15.7 | 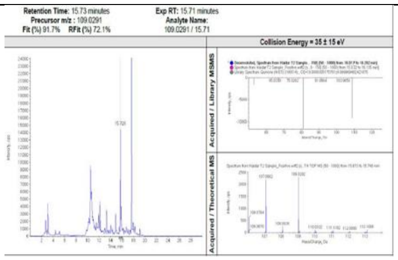  | 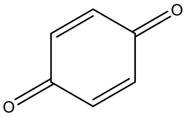  Quinone |
| Trimethoxyresveratrol | 270 | 2.09 | 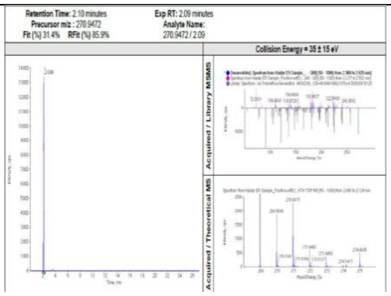  | 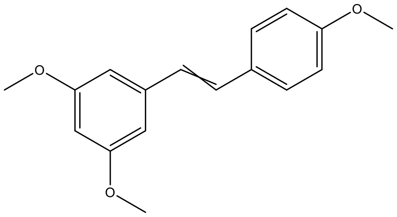  |
| Catechin | 290 | 2.3 | 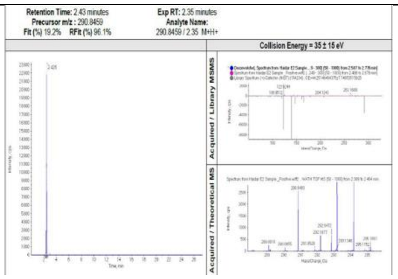  | 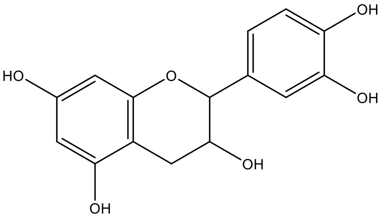  Catechin |
| 4-Allyloxycoumarin | 203 | 15.98 | 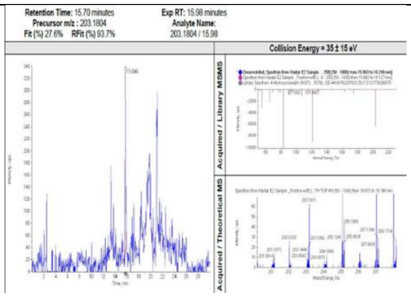  | 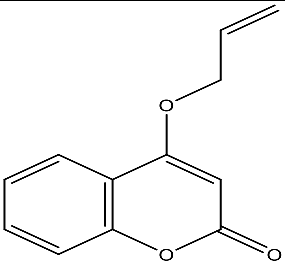  4-Allyloxycoumarin |

### 3.3.1Preliminary phytochemical investigation of different fractions through GC-MS

Preliminary phytochemical screening of the tested fractions for the presence of flavonoids, phenols, alkaloids, coumarin, cardiac glycoside and terpenoids.

**Table s3:** Qualitative analysis of phytochemical constituents in different fractions of plant parts.

| Part used | fraction | flavonoid | alkaloid | phenol | saponnin | Sterols and/or triterpenes |
|---|---|---|---|---|---|---|
| root | Petroleum ether | - | - | - | - | ++ |
| | Chloroform | + | + | + | + | + |
| | Ethyl acetate | ++ | - | ++ | + | - |
| Aerial parts | Petroleum ether | - | - | - | - | + |
| | Chloroform | ++ | +++ | + | + | - |
| | Ethyl acetate | +++ | ++ | ++ | + | - |
| (+++) Strong presence; (++) Moderately present; (+): Slightly present; (-): Negative test | | | | | | |

**GC-MS result of petroleum ether in aerial parts and root**

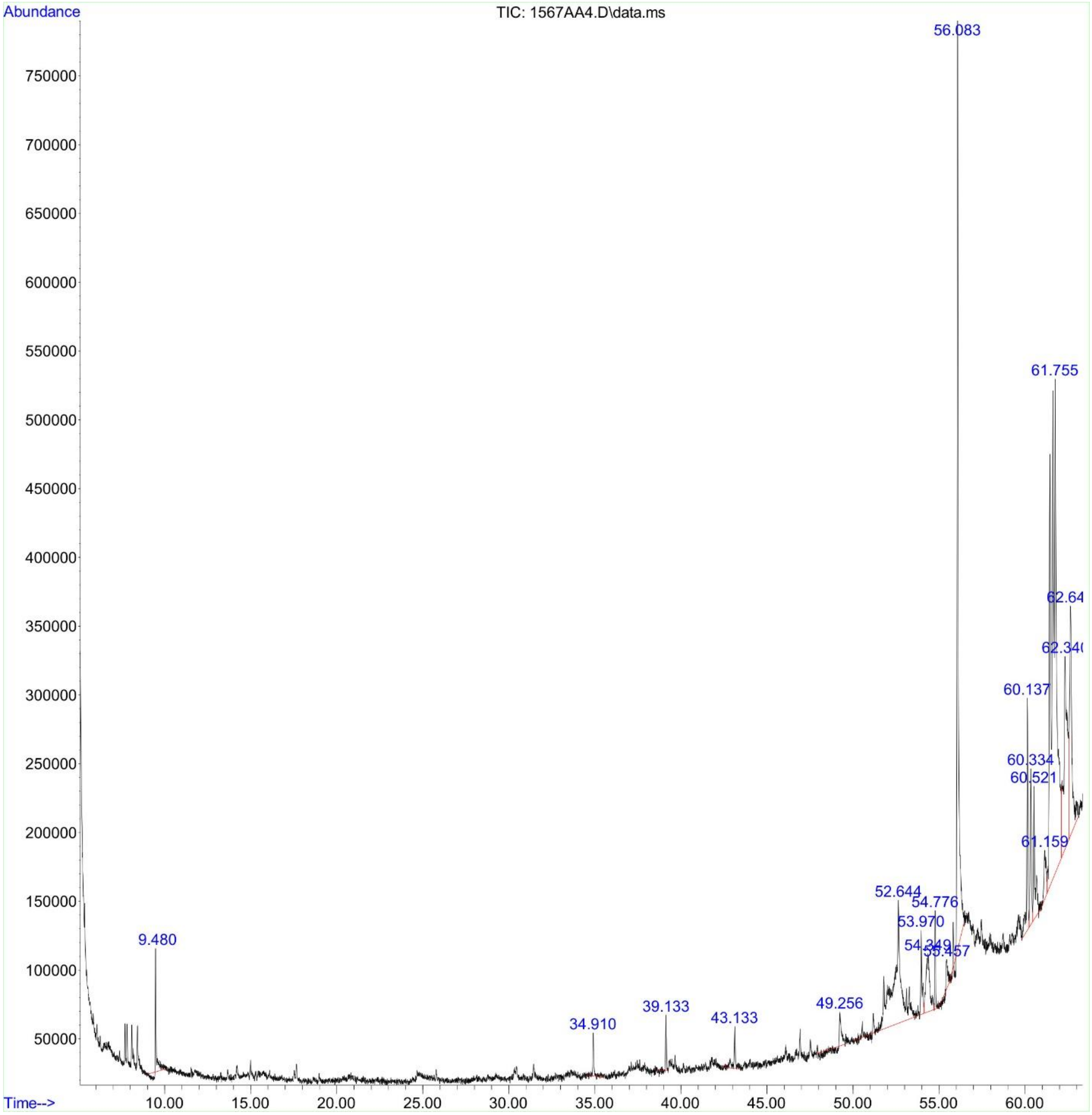


**Figure s3:GC-MS diagram of petroleum ether of aerial parts**

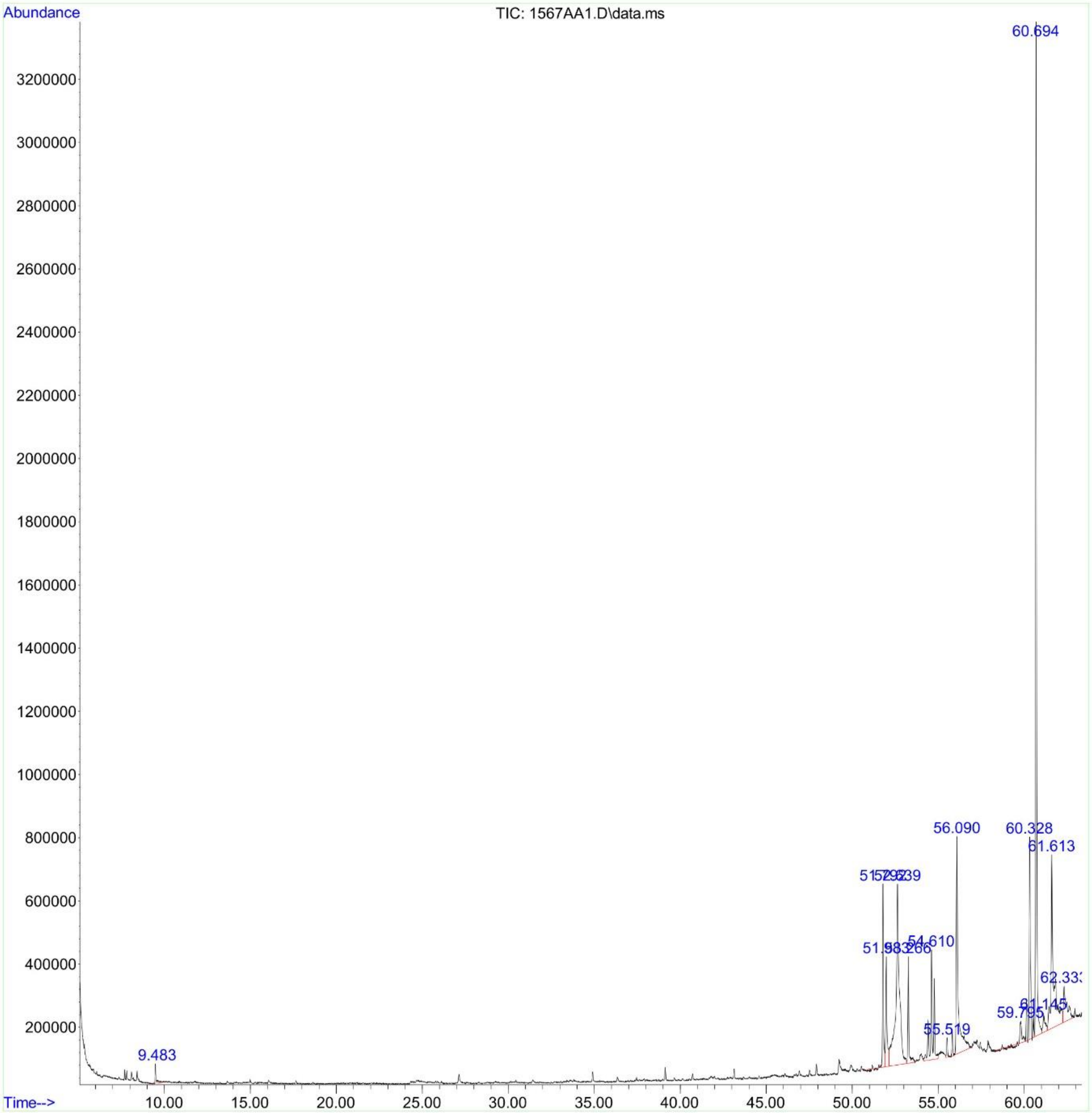


**Figure s4:** GC-MS diagram of petroleum ether of roots

**Table s4:** Compounds identified in Root in petroleum ether fraction

| peak | RT | Name | Cas number | quality |
|---|---|---|---|---|
| **Root** pet. ether fraction | | | | |

| | | | | |
|---|---|---|---|---|
| 1 | 9.482 | 4-hydroxy-4-methyl- 2-Pentanone | 123-42-2 | 78 |
| 2 | 34.910 | Tetradecane | 629-59-4 | 96 |
| 3 | 39.133 | pentadecane | 629-62-9 | 98 |
| 4 | 43.134 | Eicosane | 112-95-8 | 96 |
| 5 | 49.255 | Myristic acid | 544-63-8 | 93 |
| 6 | 52.644 | Phthalic acid, hexyl tetradecyl ester | 849-07-0 | 64 |
| 7 | 53.970 | Nonadecane | 629-92-5 | 96 |
| 8 | 54.348 | Tetracosane | 646-31-1 | 97 |
| 9 | 54.776 | Hexadecanoic acid, methyl ester | 112-39-0 | 98 |
| 10 | 55.456 | cis-9-Tetradecenoic acid, propyl ester | 427-94-7 | 60 |
| 11 | 56.085 | n-Hexadecanoic acid | 57-10-3 | 99 |
| 12 | 60.137 | 9,12-Octadecadienoic acid (Z,Z)-methyl ester | 112-63-0 | 99 |
| 13 | 60.331 | 9-OCTADECENOIC ACID | 2027-47-6 | 66 |
| 14 | 60.520 | 9-Octadecenoic acid, methyl ester | 1937-62-8 | 95 |
| 15 | 61.160 | trans-3,3a,4,5,6,7-Hexahydro-7thienyl)cyclohexa[c]isoxazole | 224-10-6 | 49 |
| 16 | 61.755 | 9-Octadecenoic acid, (E)- | 12-79-8 | 98 |

| | | | | |
|---|---|---|---|---|
| 17 | 62.338 | (+-)-1-Ethoxy-r-1-phenyl-c-2-fluorobenzocycloheptane | 479-78-3 | 86 |
| 18 | 62.640 | HENICOSANE | 629-94-7 | 95 |

**Table s5:** Compounds identified in Aerial parts in petroleum ether fraction

| **Airial** parts pet. Ether fraction | | | | |
|---|---|---|---|---|
| 1 | 9.482 | 4-hydroxy-4-methyl- 2-Pentanone | 123-42-2 | 78 |
| 2 | 51.793 | Neophytadiene | 504-96-1 | 99 |
| 3 | 51.981 | 2-Pentadecanone, 6,10,14-trimethyl | 502-69-2 | 96 |
| 4 | 52.639 | Hexanedioic acid, bis(2-ethylhexylester | 103-23-1 | 60 |
| 5 | 53.267 | 2-Hexadecen-1-ol, 3,7,11,15-tetramethyl-, [R-[R*,R*-(E)]]- | 150-86-7 | 87 |
| 6 | 54.611 | 14-.BETA.-H-PREGNA | 494-06-1 | 49 |
| 7 | 55.519 | PHYTOL ISOMER | 519-74-6 | 74 |
| 8 | 56.091 | n-Hexadecanoic acid | 57-10-3 | 99 |
| 9 | 59.794 | Tetratriacontyl heptafluorobutyrat | 2000940-33-6 | 42 |
| 10 | 60.326 | (9,12,15-Octadecatrienoic acid, methyl ester, (Z,Z,Z)- | 301-00-8 | 99 |

| | | | | |
|---|---|---|---|---|
| 11 | 60.692 | Phytol | 150-86-7 | 91 |
| 12 | 61.143 | Methyl stearate | 112-61-8 | 92 |
| 13 | 61.612 | 9,12,15-Octadecatrienoic acid | 463-40-1 | 93 |
| 14 | 62.332 | Thiosulfuric acid (H2S2O3), S-(2-aminoethyl) ester | 2937-53-3 | 78 |

## GC-MS diagram of petroleum ether of roots

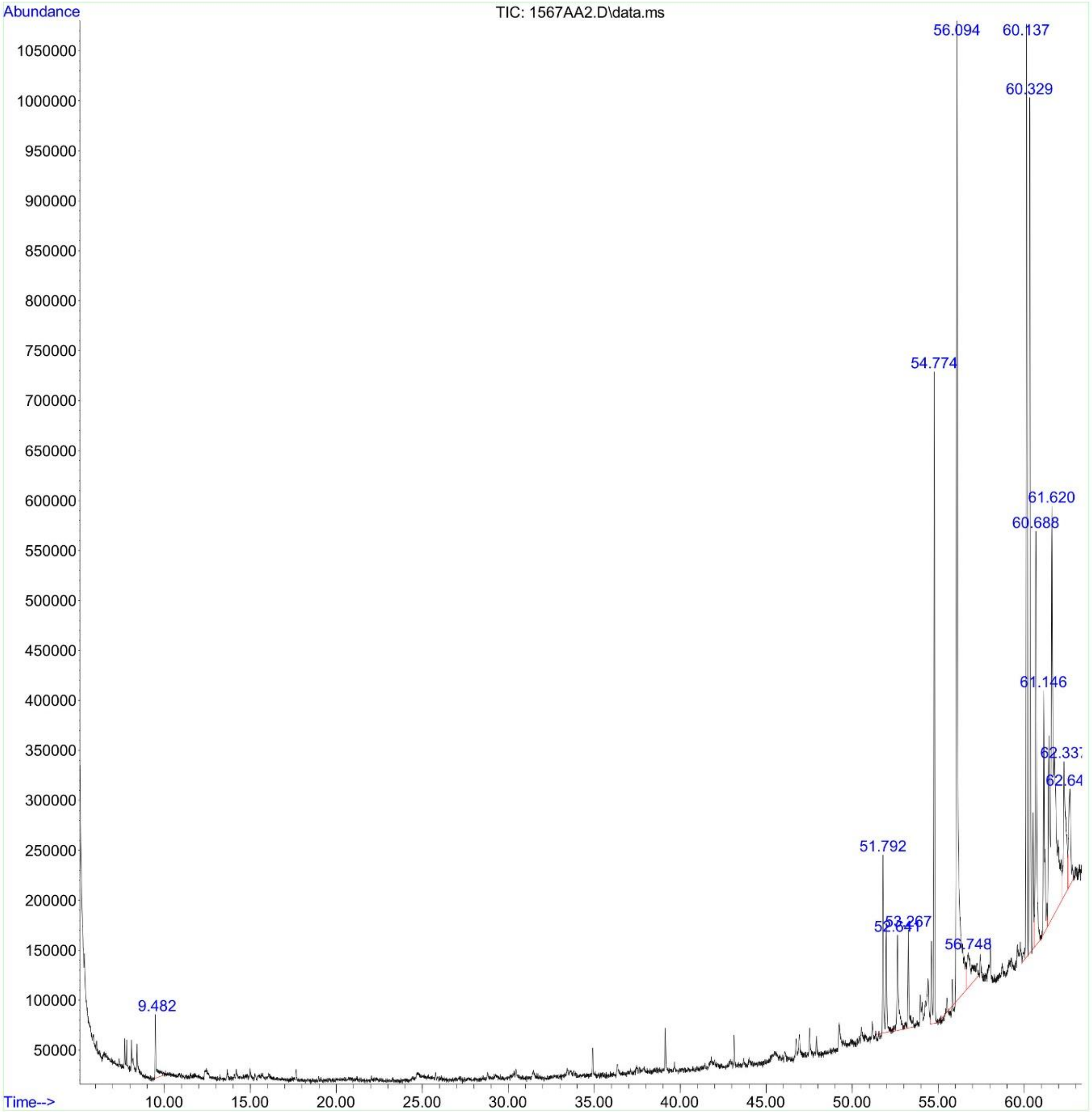


**Figure s5: GC-MS diagram of chloroform fraction of aerial parts**

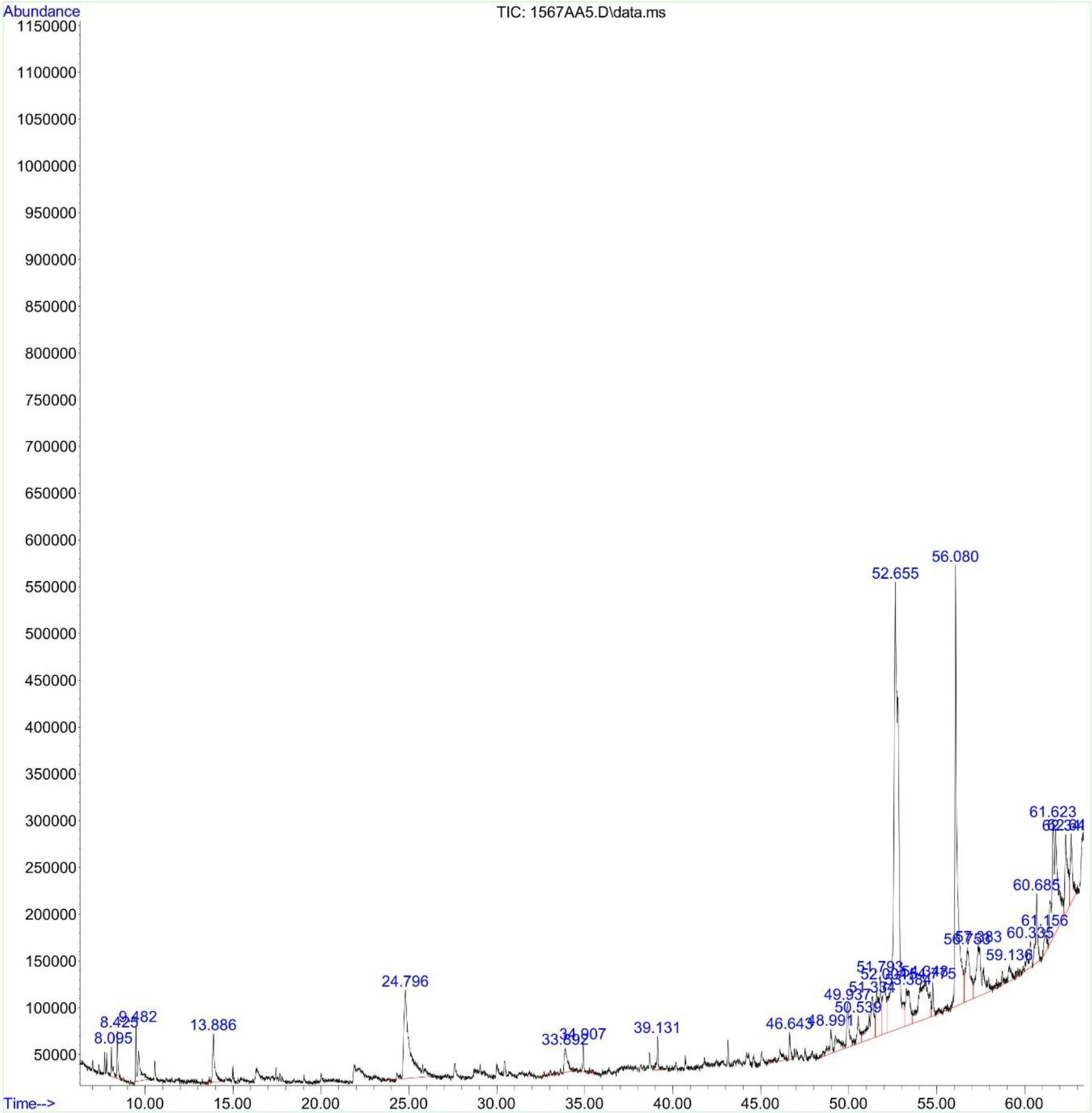


**Figure s6: GC-MS diagram of chloroform fraction of roots**

**Table s6:** Compounds identified in chloroform fraction of the aerial parts and the roots

| **Aerial parts** chloroform fraction | | | | |
|---|---|---|---|---|
| 1 | 8.094 | 3-Hexanol | 623-37-0 | 78 |

| | | | | |
|---|---|---|---|---|
| 2 | 9.482 | 4-Hydroxy-4-methyl-2-pentanone | 123-42-2 | 96 |
| 3 | 34.910 | Tetradecane | 629-59-4 | 98 |
| 4 | 39.133 | Pentadecane | 629-62-9 | 96 |
| 5 | 43.134 | Hexadecane | 544-76-3 | 93 |
| 6 | 52.702 | 1,4-bis(2-ethylhexyl) ester1,4-Benzenedicarboxylic acid | 6422-86-2 | 64 |
| 7 | 54.365 | butyl hexadecyl esterSulfurous acid | 67845-93-6 | 96 |
| 8 | 54.776 | methyl ester Hexadecanoic acid | 112-39-0 | 97 |
| 9 | 56.079 | n-Hexadecanoic acid | 57-10-3 | 98 |
| 10 | 56.748 | (1R,4R)-1-methyl-4-(6-Methylhept-5-en-2-yl)cyclohex-2-enol | 82769-01-5 | 60 |
| 11 | 57.228 | 1-(1,5-dimethylhexyl) -4-(4-methylpentyl)-Cyclohexane | 31906-04-4 | 99 |
| 12 | 60.663 | 6-methyl-1H-Indole | 3420-02-8 | 99 |
| 13 | 61.183 | 3'-hydroxy-5'-propylphenyl 2,4-dihydroxy-6-pentylbenzoate | 2524-37-0 | 66 |
| 14 | 61.635 | N-[4-bromo-n-butyl]- 2-Piperidinone | 195194-80-0 | 95 |

| | | | | |
|---|---|---|---|---|
| 15 | 62.338 | Thiosulfuric acid (H2S2O3), S-(2-aminoethyl) ester | 2937-53-3 | 49 |
| 17 | 62.641 | Eicosane | 112-95-8 | 98 |

| peak | RT | Name | Cass number | |
|---|---|---|---|---|
| **Root** chloroform fraction | | | | |
| 1 | 8.094 | (1E)-2-PROPENAL DIMETHYLHYDRAZONE | 13466-39-2 | 78 |
| 2 | 8.425 | Hexamethyl-Cyclotrisiloxane | 541-05-9 | 96 |
| 3 | 9.482 | 4-Hydroxy-4-methyl-2-pentanone | 123-42-2 | 98 |
| 4 | 13.883 | 3-methyl-Pentanoic acid | 203-297-7 | 96 |
| 5 | 24.794 | Benzoic acid | 65-85-0 | 93 |
| 6 | 33.892 | p-Methoxybenzylazidoformate | 25474-85-5 | 64 |
| 7 | 34.910 | Tetradecane | 629-59-4 | 96 |
| 8 | 39.133 | Pentadecane | 629-62-9 | 97 |
| 9 | 46.643 | N-(4-methoxyphenyl)-Pentanamide | 182887-95-2 | 98 |
| 10 | 48.992 | p-Methoxybenzylazidoformate | 25474-85-5 | 60 |
| 11 | 49.935 | 5,6,7,7a-tetr2(4H)-Benzofuranone | 17092-92-1 | 99 |

| | | | | |
|---|---|---|---|---|
| 12 | 50.541 | chloro-8-methyl-8-Azabicyclo[3.2.1]octane | 51275-31-1 | 99 |
| 13 | 51.336 | 1H-Indole-3-acetonitrile | 771-51-7 | 66 |
| 14 | 51.793 | Nonacosane | 630-03-5 | 95 |
| 15 | 52.004 | Eicosane | 112-95-8 | 49 |
| 16 | 52.656 | bis(2-) ester Hexanedioic acid | 103-23-1 | 98 |
| 17 | 53.382 | beta.-Sitosterol acetate | 915-05-9 | 86 |
| 18 | 54.348 | Eicosyl isopropyl ether | 108-20-3 | 95 |
| 19 | 54.776 | methyl ester Hexadecanoic acid | 112-39-0 | 78 |
| 20 | 56.079 | n-Hexadecanoic acid | 57-10-3 | 96 |
| 21 | 56.754 | 4-methyl-.beta-methylene-, trans-Cyclohexaneethanol, | 6236-88-0 | 98 |
| 22 | 57.382 | cis-p-Menth-8(10)-en-9-ol | 5502-99-8 | 96 |
| 23 | 59.137 | Methyl (6Z)-7-(3-Furyl)hept-6-enoate | 1745-17-1 | 93 |
| 24 | 60.337 | Octadecanenitrile | 638-65-3 | 64 |
| 25 | 60.686 | 3,7,11,15-tetramethyl-, [R-[R*,R*-(E)]]- 2-Hexadecen-1-ol | 7541-49-3 | 96 |
| 26 | 61.154 | 2-Methyl-Z,Z-3,13-octadecadienol | | 97 |
| 27 | 62.343 | Octadecanoic acid | 57-11-4 | 98 |

| | | | | |
|---|---|---|---|---|
| 28 | 62.640 | Octacosane | 630-02-4 | 60 |

**Table s7.** Antioxidant Activity in DPPH Assay.

| Concentration (mg/mL) | Leaves (%)±SD | Roots (%)±SD | Rutin (%)±SD | Astragalin ± SD(%) | Resveratrol (%)±SD |
|---|---|---|---|---|---|
| 1.0 | 75.6+2.7799 | 97.3+1.0189 | 84.1+0.6792 | 11.8+4.6690 | 77.8+4.6690 |
| 0.1 | 46.8+3.1126 | 94.4+0.3396 | 93.0+2.0658 | 4.9+2.9017 | 84.4+4.08 |
| 0.01 | 45.1+3.0186 | 58.3+2.0377 | 53.4+0.74 | 6.8+5.35 | -40.8+5.35 |
| 0.001 | 36.5+6.7839 | 43.0+6.4527 | 35.0+2.2270 | 6.9+3.63 | -75.9+2.63 |

**Table s8. Antioxidant activity in ABTS assay**

| Concentration (mg/mL) | Leaves (%)±SD | Roots (%)±SD | Rutin (%)±SD | Astragalin ± SD(%) | Resveratrol (%)±SD |
|---|---|---|---|---|---|
| 1.0 | 37±4.41 | 88±0.0 | 78±4.21 | 25±2.88 | 92±1.57 |
| 0.1 | 10±9.27 | 31±8.60 | 11±5.10 | 13±6.26 | 50±0.63 |
| 0.01 | -5±18.15 | 7±11.93 | -5±9.28 | 14±0.70 | 13±14.28 |
| 0.001 | 6±10.81 | 11±4.45 | 3±9.41 | 16±0.53 | 9±2.86 |

**Table s9. Antioxidant activity in FRAP assay**

| Concentration (mg/mL) | Leaves (%)±SD | Roots (%)±SD | Rutin (%)±SD | Astragalin (%)±SD | Resveratrol (%)±SD |
|---|---|---|---|---|---|
| 1.0 | 34.48±1.09 | 82.35±0.13 | 47.59±0.28 | 12.14±1.80 | 61.62±0.29 |
| 0.1 | 1.94±2.88 | 36.67±1.08 | 13.14±3.12 | 1.94±0.37 | 19.15±1.33 |
| 0.01 | -1.33±2.38 | 3.80±1.52 | 1.94±1.91 | -0.66±4.68 | 5.59±3.21 |
| 0.001 | -2.70±1.80 | -1.33±4.26 | -1.33±1.35 | -0.66±1.18 | 0.65±3.19 |

**Table s10.** Antioxidant activity in CUPRAC assay.

| Concentration (mg/mL) | Leaves (%)±SD | Roots (%)±SD | Rutin (%)±SD | Astragalin (%)±SD | Resveratrol (%)±SD |
|---|---|---|---|---|---|
| 1.0 | 71.50±1.85 | 95.62±0.00 | 94.40±3.28 | 87.11±2.08 | 93.54±1.06 |
| 0.1 | 29.44±4.63 | 85.48±0.59 | 77.96±0.24 | 47.92±1.68 | 77.30±1.23 |
| 0.01 | -20 ±3 | 52.45±2.17 | 37.28±2.68 | 14.63±1.34 | 43.73±3.94 |
| 0.001 | -33 ±7 | 3.85±1.37 | -22 ±13 | -10 ±4 | 4.89±4.81 |

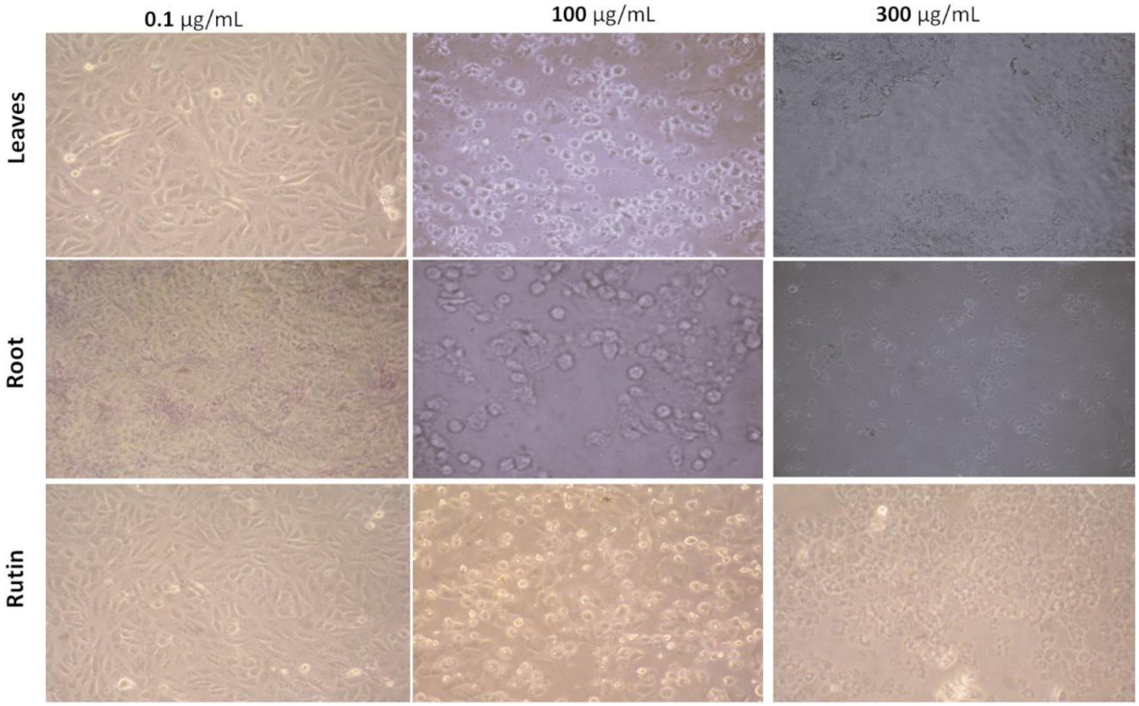


**Figure s7A: activity of Leaves, roots, and rutin on HCT-116 cells line**

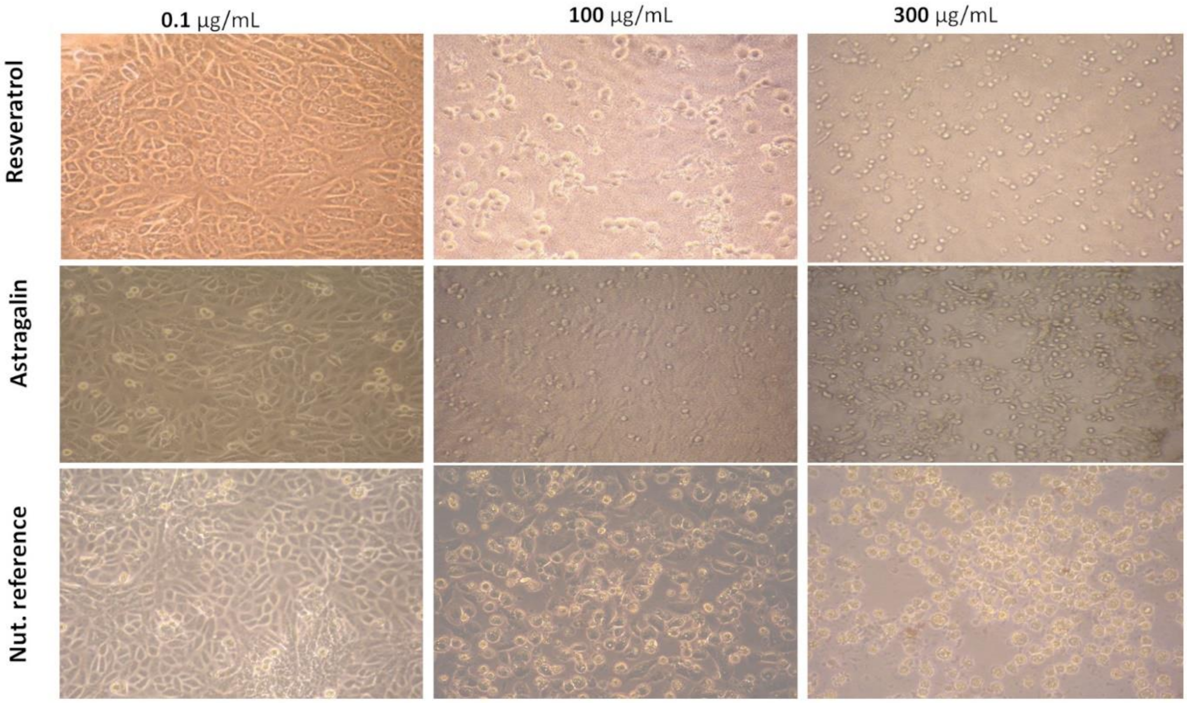


**Figure s7B: activity of resveratrol , astragalin, and Nutlin ( reference compound) on HCT-116 cells line**
